# Characterizing the ecological niche of insertion sequences within prokaryotic genomes

**DOI:** 10.1093/ismejo/wrag159

**Published:** 2026-07-11

**Authors:** Flora Gaudillière-Jami, Sophie Abby, Thomas Hindré, Ivan Junier

**Affiliations:** TIMC, UMR 5525, VetAgro Sup, Grenoble INP, CNRS, Université Grenoble Alpes, Grenoble 38000, France; TIMC, UMR 5525, VetAgro Sup, Grenoble INP, CNRS, Université Grenoble Alpes, Grenoble 38000, France; TIMC, UMR 5525, VetAgro Sup, Grenoble INP, CNRS, Université Grenoble Alpes, Grenoble 38000, France; TIMC, UMR 5525, VetAgro Sup, Grenoble INP, CNRS, Université Grenoble Alpes, Grenoble 38000, France; Centre de Biologie Intégrative (CBI), Université de Toulouse, CNRS, UPS, F-31000 Toulouse, France

**Keywords:** ecology of transposons, genome ecology, genome evolution, prokaryotic genomes, transposable elements

## Abstract

Insertion sequences (ISs) are widespread prokaryotic transposable elements, often regarded as genomic parasites that primarily cause deleterious mutations. However, they can also promote adaptive changes. These antagonistic properties make their overall impact on prokaryotic evolution difficult to grasp. Here, we address this challenge by leveraging the framework of transposon ecology to analyze IS occurrences across and within 30 499 prokaryotic genomes. Combining phylogenomics with multi-scale genomic analysis, quantitative ecology, and mathematical modeling, we provide evidence that although genomes generally provide sufficient resources for IS coexistence, universal mechanisms shape their occurrence and chromosomal distribution across genomes. These include (i) the preferential localization of ISs within highly variable and GC-heterogeneous chromosomal regions of genomic plasticity, which act as the primary reservoir of IS niches; (ii) a linear scaling between IS abundance and niche size, with an average of *5.4* additional accessible insertion sites per IS; (iii) a dependence of IS occurrence on the presence of other ISs, suggesting a form of group behavior; (iv) the accumulation of AT-rich sequences in both coding and noncoding regions up to 100 kb around ISs, indicative of ecological isolation; and (v) the spatial partitioning of mobile genetic elements around ISs, reminiscent of ecological niche differentiation. Besides these general principles, we also uncover niche specificities associated with particular IS families, hinting at regulatory mechanisms that modulate IS activity. Altogether, this comprehensive transposon ecology approach offers new insights and avenues for understanding IS–host interactions and genome evolution, moving beyond traditional host-centric perspectives.

## Graphical Abstract

**Graphical Abstract:**
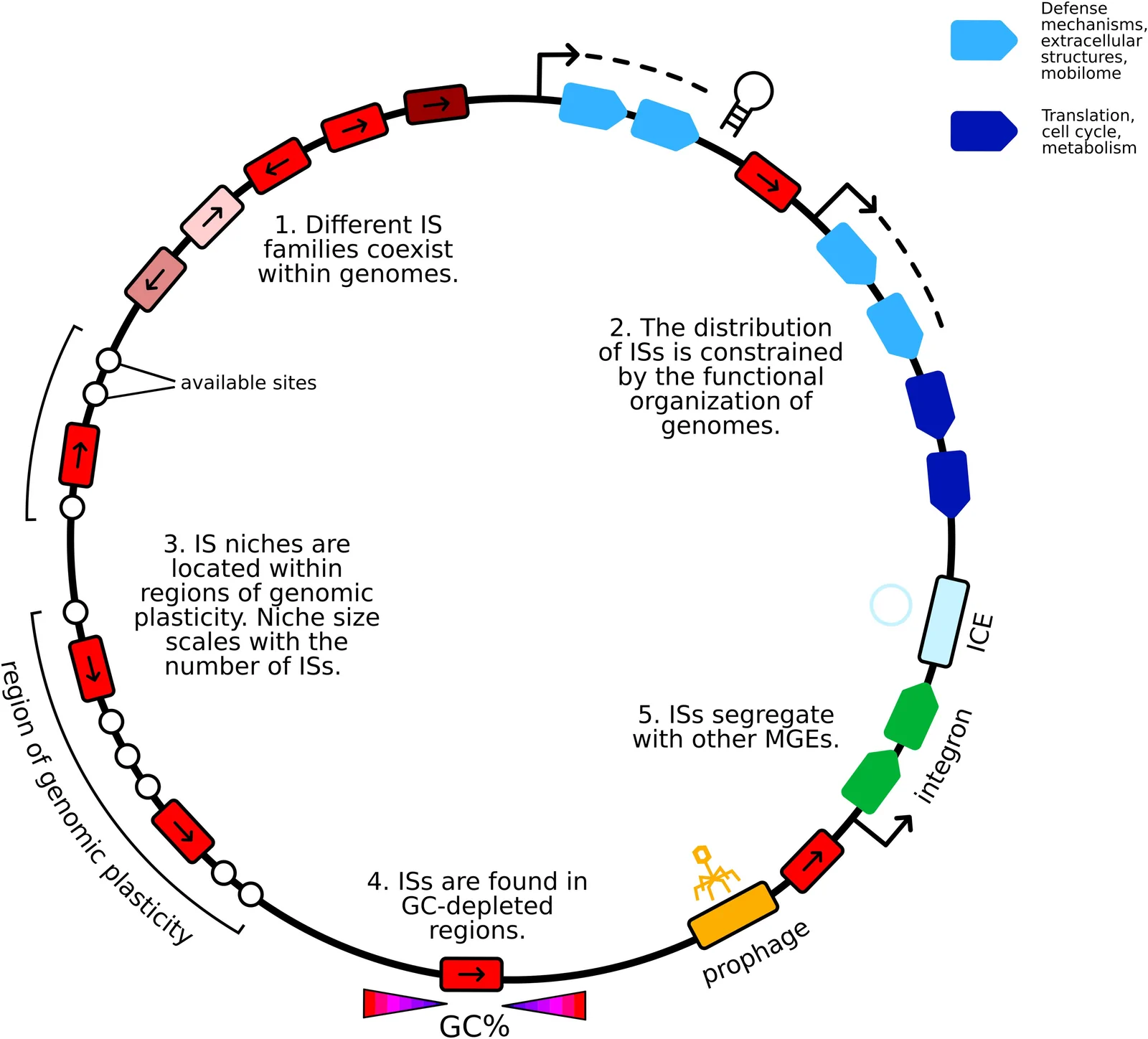

## Introduction

Insertion sequences (ISs) are the smallest transposable elements (TEs) found in prokaryotic genomes [1, 2]. They can move within a genome by either copy-paste or cut-and-paste transposition, and can be transferred between hosts via intercellular mobile genetic elements (MGEs), such as plasmids. Upon transposition, they generate structural variations within genomes, making them key contributors to genomic plasticity and evolvability [3]. They can insert into genes and disrupt their expression [2] as well as cause large-scale genomic deletions and inversions [4]. These genomic changes are often deleterious to host fitness, which is why ISs have often been viewed as genomic parasites. However, they can occasionally generate beneficial mutations, for instance by knocking out costly genes [5], modulating functional elements such as gene promoters [6, 7], or facilitating the horizontal gene transfer (HGT) of antibiotic resistance genes [8].

These antagonistic properties of ISs—being often detrimental yet sometimes offering evolutionary advantages—combined with their highly dynamic nature make their overall impact on prokaryotic evolution and genome biology difficult to grasp. Consequently, the frequent occurrence of ISs within chromosomes [9] raises questions about the factors influencing their genomic location and maintenance [1]. Several of these factors have already been reported. At the local scale, several ISs are known to recognize specific DNA motifs [10, 11], whereas others show sensitivity to the AT richness of the targeted regions [1]. At the chromosome scale, a mutation accumulation study in *Escherichia coli* has shown that ISs tend to insert preferentially into noncoding regions [11]. This study also reported that insertions often occur near preexisting copies of the same family. Additionally, a comparative study of 80 bacterial species revealed that about 20% of IS copies are located in chromosomal hotspots representing a small fraction (1%) of chromosomes and corresponding to recent HGT [12].

Altogether, these observations raise fundamental, unresolved questions, including (i) How can we explain the widespread presence of ISs across prokaryotic genomes? (ii) Which genomic features of chromosomes constrain the evolutionary maintenance of ISs? (iii) To what extent does this maintenance depend on the characteristics of the IS itself? To tackle these questions, a promising avenue comes from the field of transposon ecology [13], where TEs are viewed as independent entities inhabiting host genomes and potentially interacting through mechanisms such as competition or cooperation [14–17]. TE-centric ecological approaches have been applied to eukaryotes, offering novel insights into the distribution and abundance of eukaryotic TEs [16, 18, 19]. In prokaryotes, the ability of ISs to self-replicate, their interactions with other MGEs, and the diversity of host–IS relationships make them ideal candidates for these approaches [1, 15, 16]. However, despite growing recognition, ecological approaches applied to ISs have remained largely anecdotal.

Here, we fill this gap and demonstrate how a comprehensive transposon ecology approach can provide novel, quantitative insights into the determinants of IS occurrence both across and within prokaryotic genomes. To this end, we treat each IS as an individual belonging to a specific species (the IS family) and interacting with its ecosystem (the host cell), whose characteristics—such as genomic GC content, gene and MGE density, and specific sequence features—can influence its ability to transpose and survive at specific loci. A central concept in ecology concerns the *ecological niche* of a species, which broadly represents the space it occupies within an ecosystem [20]. By analogy with the environmental niche, defined as the set of conditions that permit a species’ long-term existence, here we define the niche of an IS as the genomic regions and the properties of the underlying DNA sequences associated with its persistence.

Using this framework, we address the following questions, difficult to formulate within a classical host-centric genomics approach: How diverse is the IS species community within a given genome, and what determines it? What are the properties of the ecological niches of ISs within genomes? Do all IS families share the same ecological niche, or is there evidence of niche specificity? To that end, we leverage the large collection of prokaryotic genome sequences available in public databases, along with automated tools for IS annotation [21, 22], analyzing the distribution of IS elements from 30 IS families across and within 30 499 complete prokaryotic genomes.

## Materials and methods

### Genome data and statistics

A total of 30 499 full prokaryotic genomes were downloaded from NCBI on 15 July 2022. We used the taxonomy and replicon classification (chromosomes/plasmid) provided in the associated metadata. The dataset includes 8106 distinct species. We accounted for sampling bias by weighting each genome by the abundance of the species (weight = 1/(nb of genomes)) and by conducting analyses using these weights. Genomes lacking species taxid information were dropped from the analysis (90 genomes). In specific analyses where weighting is cumbersome, we considered a single strain per species.

### Detection of ISs

ISs were detected using the digIS software (https://github.com/janka2012/digIS) [22], which requires only the fasta files of genomes—a cross-validation was performed by comparing digIS outputs with those of ISEScan [21] for a subset of 21 298 genomes (see Supplementary Note 3). From a total of 1 502 064 reported potential ISs, we retained 993 902 ISs reported as complete elements. Among these, 770 751 elements were found on chromosomes, 153 409 on plasmids, and 69 742 on unclassified replicons. These elements fall into 30 families, corresponding to the ISfinder classification [23]. Some detected IS elements matched several HMM models: when the models belonged to the same family, they were classified into it; otherwise, they were labeled as “other” and excluded from our analysis of family-dependent properties.

### Rank abundance curve and ecological models

We considered two generic ecological models—lognormal and power law—to fit the rank abundance curve (RAC). The expected abundance *f(r)* for a given rank *r* in these models is given by (i) $f(r) = F^{-1}_{\mu ,\sigma }(1-\frac{r}{S})$ for the lognormal model, where $F_{\mu ,\sigma }$ is the cumulative density function of the lognormal distribution with mean *μ* and standard deviation *σ*, *S* is an effective maximum number of species, and (ii) $f(r)=Ar^{-\alpha }$ for the power-law model. The model parameters (*μ*, *σ*, and *S* for the lognormal model; *A* and *α* for the power-law model) were estimated by least-squares minimization.

### Identification of insertion context

The 200 bp flanking ISs on each side were extracted and joined into a single sequence, which was subsequently searched against the NCBI prokaryotic sequence database using MegaBLAST (with an e-value threshold set at 1e-60 and max_target_seqs at 5). When a hit sequence was available in our database, the annotation file for that sequence was examined to see if the hit position was annotated as a coding sequence (CDS). If so, the IS was classified as having interrupted a gene. Otherwise, it was classified as an intergenic transposition.

### Identification and analysis of genes flanking intergenic ISs

For each detected IS (except ISs classified as intragenic), we retrieved the flanking genes as the closest upstream and downstream genes using NCBI metadata annotation tables. To avoid issues related to potentially incomplete genome annotations, only ISs with flanking genes located <1 kb away were considered for the analysis of gene orientation and synteny (640 578 ISs out of 993 902).

### Functional annotation of genes using COG categories

The functional classification of interrupted and flanking genes was performed using COGclassifier (https://github.com/moshi4/COGclassifier), which classifies genes based on sequence similarity to representative orthologs. We retained 22 of the 26 COG categories [24], excluding categories A, B, Y, and Z (eukaryote-specific).

### Operon database and gene synteny

*Escherichia coli* K12 MG1655 operons were retrieved from RegulonDB v12.0 [25]. Genes in a synteny relationship were identified on the basis of their COG annotation (see above), using a previously established list of COG pairs identified to be in synteny relationships [26]. This list is provided in Supp. Table 2.

For the synteny analysis of gene pairs from distinct operons flanking an IS, we considered only those pairs that occur as neighbors in at least one other *E. coli* genome.

### Identification of terminator-like structures

To identify potential Rho-independent intrinsic terminators around ISs, we scanned upstream and downstream for inverted repeats followed by T-tracks. Based on structural analyses [27, 28], we considered inverted repeats with gap sizes ranging from 3 to 7 (hairpin loop size in bases) and arm lengths from 4 to 14 (stem size in base pairs), and T-tracks composed of >4 Ts. The terminator analysis was conducted around complete ISs (as defined by digIS) and around IS CDSs (identified by intersecting digIS coordinates with NCBI CDS annotations). For comparison, we also scanned sequences flanking randomly sampled CDSs from the genomes of our dataset.

### Identification of IS clusters

Chromosomal ISs were considered part of the same cluster if they were adjacent along the DNA and separated by <2.5 kb, the typical maximum distance observed to flanking CDSs (Fig. S4A). To mitigate false negatives, when two ISs were not immediate neighbors but were separated by <2.5 kb with a transposase annotated in-between them (despite not appearing in our dataset), the two ISs were considered to be part of the same cluster. We also verified that our findings do not depend on this assumption.

### *Escherichia coli* phylogenetic tree

To examine the dynamics of a specific IS cluster in *E. coli*, we focused on one conserved gene pair (as defined by PpanGGOLIN; see below) and randomly sampled 50 genomes containing an IS cluster between these genes, along with 30 genomes devoid of IS at that locus. A phylogenetic tree was then built for these 80 genomes using 41 phylogenetic markers selected with the markerfinder tool (see methodology described in [29]), with a *Xanthomonas oryzae* genome (GCA_001928215.2_ASM192821v2) used as an outgroup to root the tree (removed on the final visualization). The tree was simplified to keep only 59 leaves.

### Persistent, accessory genes and regions of genome plasticity

Persistent, shell, and cloud genes were identified using the PpanGGOLiN tool [30] (https://github.com/labgem/PpanGGOLiN). For each chromosome in the genomes of the three analyzed species (*E. coli* (GC%=50.7), *Pseudomonas aeruginosa* (GC%=66.2), and *Acinetobacter baumannii* (GC%=39.1)), PpanGGOLiN reports segments classified as regions of genomic plasticity (RGPs). Chromosomes were then divided into 1000 bins, and the probability to be in an RGP across all chromosomes was computed for each bin.

### Profiles of IS occurrences along a normalized chromosome

For the three species *E. coli, P. aeruginosa*, and *A. baumannii*, containing, respectively, 1917, 451, and 444 genomes in our dataset, chromosomes were first reoriented so that the origin of replication (*oriC*) corresponds to position 0, with the *rplS* gene always located on the right replichore. Second, for each chromosome, IS positions and other genomic features (RGPs, persistent genes, and so on) were normalized to chromosome length, such that position 0.5 corresponds to the replication terminus (*ter*) and position 1 loops back to *oriC*. To avoid overcounting IS occurrences inherited from a common ancestor, those with identical flanking genes and orientation were weighted so that their total contribution summed to 1.

In this context, the profiles for IS location, RGP, persistent gene location, and GC content were computed by dividing chromosomes into 1000 bins (*∼*5, 4, and 6.5 kb resolution for *E. coli, A. baumannii*, and *P. aeruginosa*, respectively). For clarity, curves were smoothed using a Gaussian filter with a standard deviation of 2 bins to reduce small-scale fluctuations, as the main variations occur at a much larger (*∼*100 kb) scale.

### GC content deviation profiles around IS positions

For all genomes in our dataset, the deviation from the average GC content around ISs was computed at 1000 positions logarithmically spaced from 1 bp to 1 Mb away from each IS, both upstream and downstream the IS with respect to its transposase CDS reading orientation. A corresponding profile was computed around an equal number of randomly selected chromosomal CDSs in each genome. Similarly, we computed the profiles associated with different genomic features (intergenic regions, CDSs, as well as first (GC1), second (GC2), and third (GC3) codon positions) by retaining only positions belonging to the investigated feature. Note that in this case, the average GC content was computed using only the positions associated with the feature. Note also that the analysis considering three groups of genomes (Fig. S16) corresponds to three distinct regimes of GC3 levels (GC1 is always higher than GC2): (i) GC3 is the lowest; (ii) GC3 is intermediate (between GC2 and GC1); and (iii) GC3 is the highest.

### Detection of MGEs

Prophage CDSs were detected using the DB-SWA tool [31] (https://github.com/HIT-ImmunologyLab/DBSCAN-SWA/). Integrative and Conjugative Elements (ICEs) and Integrative Mobilizable Elements (IMEs) were detected using the CONJScan (v2.0.1) module of MacSyFinder (v2.1.3) [32, 33] (https://github.com/macsy-models/CONJScan). Integrons were detected using IntegronFinder [34] (https://github.com/gem-pasteur/Integron_Finder). For each of these categories, a mask was applied to the genomes to consider only relevant positions when analyzing the frequency and characteristics of each type of MGE around IS positions. To make sure masks were exclusive, we defined ICE annotation as superseeding integron annotation, integron annotations as superseeding prophage annotations, and prophage annotations as superseeding IS annotations. We also verified that our results do not depend on sequences with ambiguous annotations (*i.e.* sequences annotated as at least two different types of MGEs.)

## Results

### Transposon ecology terminology

Ecological concepts and quantities for transposons have mainly been discussed and analyzed in eukaryotes. Drawing on previous work [15, 35], we therefore begin by clarifying the ecological concepts associated with prokaryotic ISs, redefining them in a genomic context:

- **Individual**: IS copy.
- **Species**: IS family as defined in the curated ISFinder database. In the ecological context, a family is understood as a lineage of ISs that share specific biochemical properties (e.g. similar transposition mechanisms, target preferences) and sequence similarity.
- **Species richness**: number of distinct IS families within a genome.
- **Population**: set of IS copies within a given genome that belong to the same family.
- **Community**: set of IS copies within a given genome, regardless of family.
- **Ecosystem**: host cell, in which an IS copy can interact with various elements including DNA sequences (e.g. host genes or other MGEs), proteins (such as host factors involved in DNA metabolism), and RNA molecules.
- **Ecological niche**: in ecology, the niche corresponds to the environmental conditions that allow the persistence of a given species. For ISs, we define the ecological niche as the range of genomic environments in which an IS can insert and persist without being lost or causing the death of the host cell. In particular, IS persistence at a locus might be influenced by a variety of genomic factors, including the function, proximity, and orientation of neighboring genes, the level of IS clustering within a genome, as well as genomic characteristics such as GC content or secondary structures.

### IS communities within genomes are well-balanced and diverse

**Figure 1.**
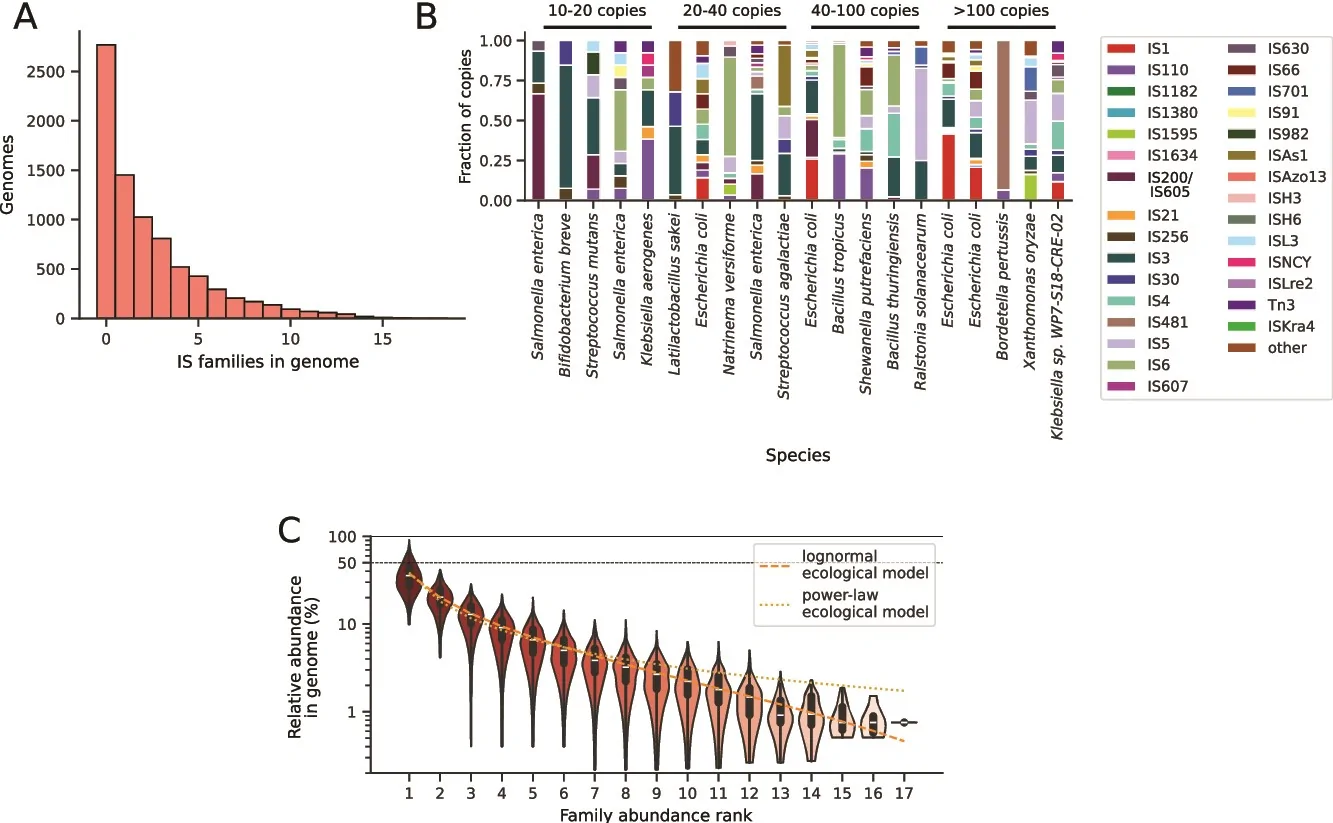
Diversity of IS communities within genomes. A) Histogram of the number of distinct IS families in prokaryotic genomes. B) IS content of 20 sample genomes from the dataset with total number of ISs ranging from 10 to 20 to >100 copies. The label ”other” refers to ISs that are not clearly attributed to a specific family (Methods). C) Best fits of the rank abundance curve of ISs using a lognormal and a power law ecological model.

Observing that 85% of genomes with at least one IS contain more than one IS family (Fig. 1A), and that multiple IS families often coexist within a given genome (Fig. 1B), we first asked whether IS families tend to coexist in similar population sizes, or whether competition leads, for instance, to the dominance of a single, fitter family. To address this, we computed the evenness of family composition (i.e. of IS species richness) within genomes using a descriptor commonly applied in ecology: the rank abundance curve (RAC). This curve provides key yet easily interpretable information about community composition by showing the proportion of species (IS families) as a function of their decreasing rank in abundance within ecosystems (host cells) [36, 37]. In particular, RACs derived from models or empirical data often fall into a few distinct shapes—typically lognormal, logseries, or power law—each reflecting different levels of evenness in species composition [38]. Our analysis for the ISs shows a RAC that is particularly well-fitted by the lognormal model (Fig. 1C), and, for instance, much less so by the power law model (Fig. 1C). This suggests a well-balanced [36, 38], mosaic nature of IS communities within prokaryotic genomes, where no single IS family dominates excessively and families rarely contribute only a small fraction of copies. In other words, provided ISs can enter a genome, the latter offers sufficient niche resources to support their coexistence.

### IS niche availability is strongly constrained by the functional organization of genomes

**Figure 2.**
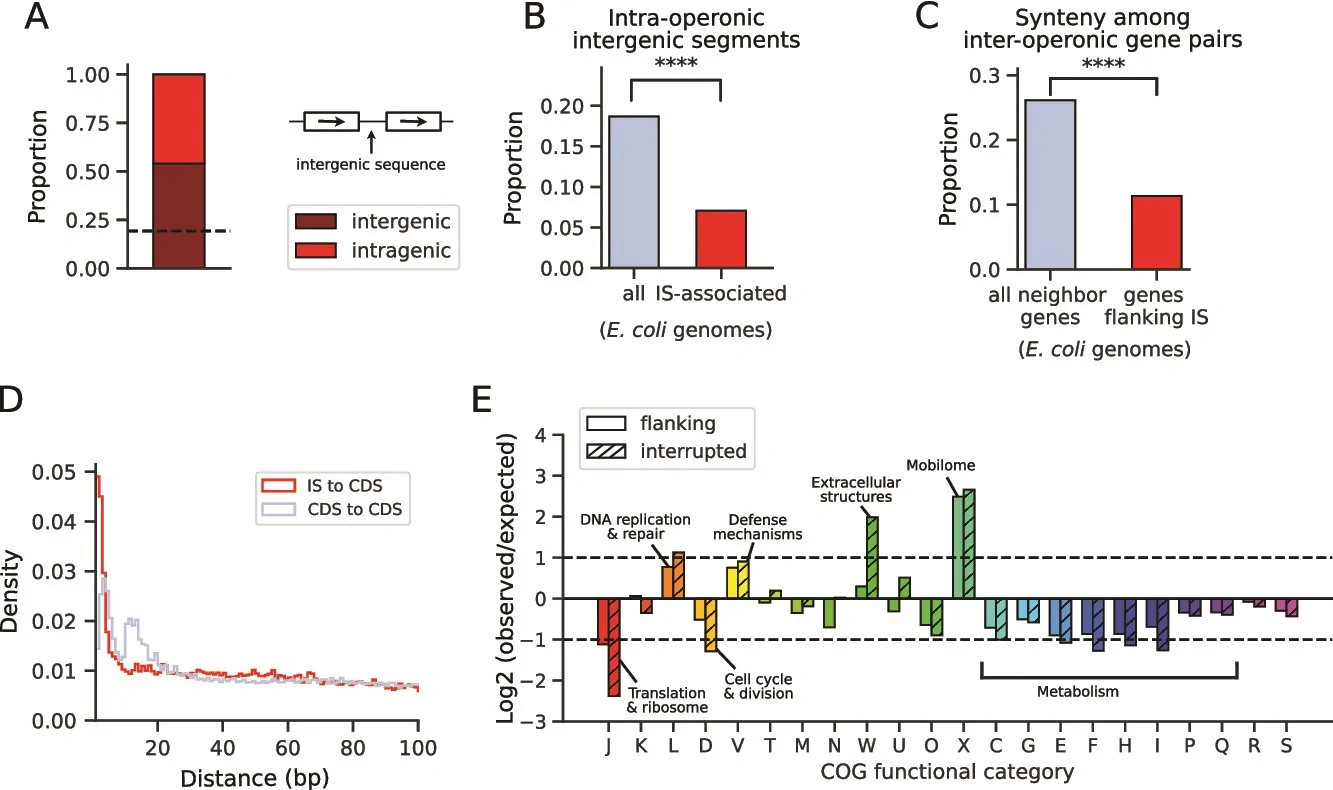
Functional environment of IS elements. A) Proportion of identifiable IS transposition sites found in intergenic segments (dark red) and intragenic segments (red). Dotted line: average proportion of intergenic sequences in prokaryotic genomes. B) In *E. coli* genomes, proportion of intergenic segments found inside of an operon (light blue) and proportion of intergenic ISs found inside of an operon (red). The statistical significance (*P < 0.001*) was computed using a chi2 test. C) In *E. coli* genomes, proportion of syntenic gene pairs among consecutive genes that belong to different operons (light blue) and proportion of syntenic gene pairs among genes flanking ISs that are found next to each other in at least one genome (red). The statistical significance (*P < 0.001*) was computed using a chi2 test. D) Normalized distribution of distances from ISs to their flanking CDSs (red) or between random CDSs (light blue), for distances ranging from 1 to 100 bp. E) Functional enrichment of genes flanking ISs (plain) or interrupted by ISs (striped). Enrichment or depletion is measured by calculating the $\log 2[f_{obs}/f_{exp}]$, with $f_{obs}$ the frequency at which a given functional category is found in either genes flanking ISs or interrupted by them, and $f_{exp}$ the frequency at which this functional category is found in randomly sampled genes. The horizontal lines indicate two-fold increase and decrease, respectively. Complementary COG annotation: K: transcription; T: signal transduction; M: cell wall/membrane; N: cell motility; U: trafficking & secretion; O: protein modification; C: energy production; G: carbohydrate metabolism; E: amino acid metabolism; F: nucleotide metabolism; H: coenzyme metabolism; I: lipid metabolism; P: ion transport; Q: secondary metabolism; R: general function prediction only; S: unknown function.

We investigated the local properties of the ecological niches occupied by ISs within host genomes by examining their immediate genomic context. Corroborating results from mutation accumulation lines in *E. coli* [11] and extending them across species, we find that ISs preferentially locate in intergenic regions rather than in regions where they interrupt coding sequences (CDSs) (Fig. 2A). This trend holds at the family level for 29 of the 30 IS families, all of which show a significant signal (Fig. S1). We then asked whether these intergenic regions are linked to specific aspects of the functional organization of genomes, which is known to be highly constrained [39, 40]. In particular, genes often belong to operons, and cofunctional operons are themselves organized into larger genomic units that tend to remain in close proximity throughout evolution, a property known as gene synteny. Analyzing *E. coli* genomes for which accurate functional genome annotations are available, and which harbor 19 of the 30 known IS families, we find that identifiable intergenic ISs are disproportionately located in inter-operonic regions (Fig. 2B), with this pattern holding for all but one IS family (Fig. S2). Furthermore, inter-operonic ISs rarely reside in-between syntenic gene pairs (Fig. 2C), with the notable exception of the IS*30*, IS*66*, and IS*91* families, which exhibit a significant opposite trend (Fig. S3).

The analysis of the location of intergenic ISs with respect to their flanking genes reveals two opposite trends. First, CDSs tend to be located farther from ISs than from random CDSs (Fig. S4A, Fig. S5). In contrast, when focusing on the occurrence of short distances below 100 bp, we find an overrepresentation of IS insertions occurring just one, two, or three base pairs upstream or downstream of CDSs (Fig. 2D), a pattern observed in 28 of the 30 IS families (Fig. S4C, Fig. S6). Finally, and consistent with recent observations [9], we observe that 17% and 50% of IS families are preferentially found between divergent and convergent gene pairs, respectively (Fig. S4B).

We examined in more detail the functions of genes interrupted by, or flanking, ISs by assigning all genes in our dataset to one of 22 bacterial Cluster of Orthologous Genes (COG) functional classes (Fig. 2E). Similar patterns are observed for interrupted and flanking genes, though functional biases are systematically stronger for interrupted genes—the only exception is a strong overrepresentation of extracellular structure functions (class W) observed only among interrupted genes; closer inspection reveals that this is largely driven by the recurrent disruption of a single gene, *fimC* involved in pilus assembly of type I fimbriae. Specifically, we observe that metabolism-associated functions globally tend to be under-represented, whereas some specific functions are strongly over-represented. These include mobilome genes (X), consistent with previously reported MGE hotspots [12], as well as defense mechanisms (V) and DNA replication and repair (L). The latter is particularly intriguing given that other essential functions, e.g. translation (J), and cell cycle (D) are strongly underrepresented.

Altogether, these results reveal a strong influence of the functional organization of genomes on IS niche availability. Much of this likely reflects purifying selection, as ISs tend to be underrepresented in regions under selective pressure. However, the overrepresentation of specific functions among IS-interrupted genes such as defense mechanisms or DNA replication and repair may instead suggest positive selection, either mediating host adaptation to new environments [41] or stress conditions [42], or possibly favoring the ISs themselves. Moreover, recurrent patterns in the local organization of ISs with respect to their flanking genes suggest that ISs often play specific roles in modulating the expression of nearby genes, as has been demonstrated experimentally in specific cases [43].

### Terminator-like sequences are overrepresented upstream of ISs

**Figure 3.**
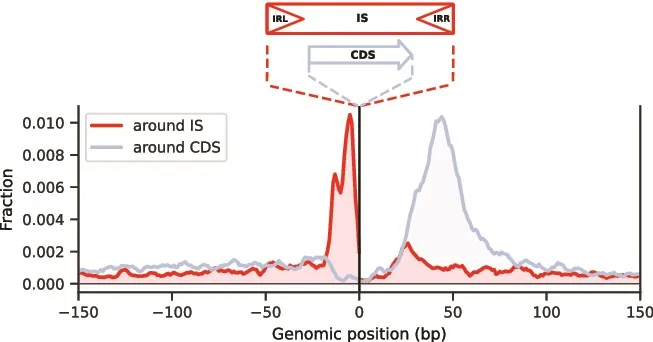
Genomic distribution of terminator-like sequences around ISs. Frequency of terminator-like sequences in the 150 bp upstream and downstream of ISs compared with the same frequency in the vicinity of random CDSs. Genomic positions are shown relative to the orientation of CDSs. The detailed signal associated with each family is provided in Fig. S7. Note that while most IS families are flanked by inverted repeats (IR) as depicted here, some families such as the IS*200*/IS*605* are not.

We observe a strong overrepresentation of DNA sequences characteristic of intrinsic transcription terminators located immediately upstream of ISs, in sharp contrast to the typical tendency of intrinsic terminators to be found downstream of genes (Fig. 3, Fig. S7)—the strongest signal is observed for the IS*200*/IS*605* family, for which experimental evidence for the activity of such terminators already exists [44]. Furthermore, in some IS families, terminator-like sequences can also be found inside of IS elements (between the limits of the IS and the transposase CDS, Fig. S8). Overall, we thus find that several IS families are disproportionately found in genomic contexts likely to limit transposase expression, which we interpret as a characteristic that promotes their persistence within genomes.

### Clustering properties: IS niche size is predicted to scale with IS number

**Figure 4.**
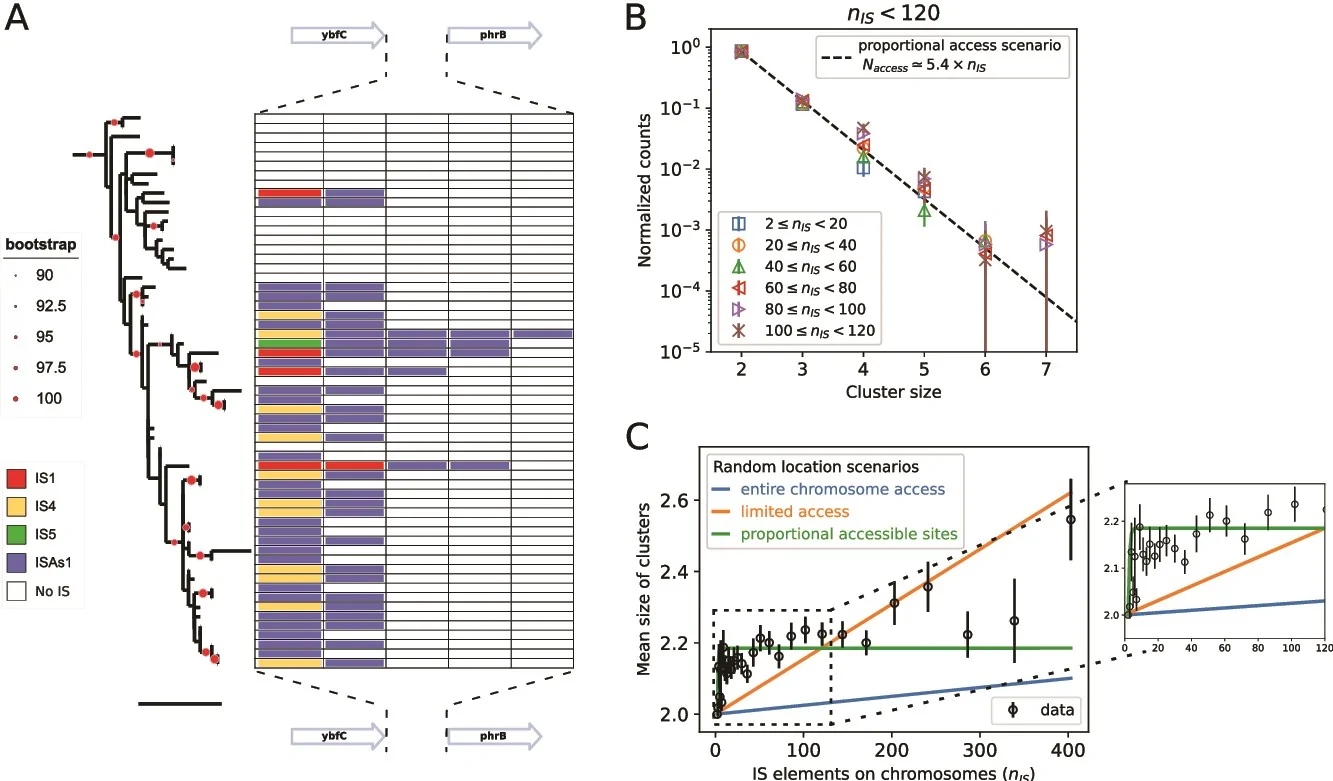
IS clusters: diversity, statistical properties, and modeling. A) Example of cluster composition diversity between two conserved genes in different *E. coli* genomes. For each genome, the IS content between the two neighboring genes *ybfC* and *phrB* is represented, ranging from 0 (empty rows) to 5 elements (fully colored row). Tree scale: 0.002 substitutions per site. B) Distribution of cluster sizes in genomes containing <120 ISs. Each plot corresponds to a distribution that has been computed by constraining the number of ISs within the genome to belong to a certain interval. The black dashed line indicates the prediction (an exponential law) of the model of proportional accessible sites, considering that each IS is associated with *∼ 5.4* accessible sites. C) Mean size of clusters as a function of the number of ISs in the corresponding chromosomes. We also report the results of three models based on random IS transpositions along the chromosome under specific constraints: (i) full accessibility, where ISs can insert and survive anywhere along the chromosome (blue); (ii) limited access, where ISs can only survive in a fixed portion of the chromosome (see Supp. Note 1 for more complex scenarios) (orange); (iii) proportional accessible sites, where the presence of an IS is associated with additional opportunities for other ISs to transpose and survive (green). For the latter scenario, the parameters are those of the model used in panel (B). We also note that part of the residual variation around the prediction of the fixed-limit scenario (ii) can be explained by an alternative scenario, in which the limited access region is not fixed and independent of chromosome size, but instead scales proportionally with it. (see Supp. Note 1). $n_{IS}$: number of ISs on the chromosome.

To better understand the behavior of ISs along chromosomes and to apprehend the nature of their niche, we investigated whether ISs tend to colocalize. To this end, we analyzed the tendency of ISs to cluster, considering ISs to be part of the same cluster if they are adjacent along the DNA and separated by <2.5 kb (Methods). In agreement with [12], we observe that in 19% of cases, an IS is part of a cluster, with cluster sizes reaching up to 18 individuals and showing diverse compositions regardless of size (Fig. S9A). The composition of a given cluster strongly varies across strains of the same species, as can be seen in *E. coli* (Fig. 4A). Therefore, chromosomal IS clusters are likely to result from a dynamical, stochastic birth-and-death-like process [45] occurring at those loci, rather than from en bloc integration of plasmid-carried IS clusters. To better apprehend this underlying process, we computed the statistical distribution of cluster sizes. We observe that for chromosomes with fewer than 120 ISs, which corresponds to 96% of our dataset, the distribution follows an exponential law with a decay constant that is independent of both the total number of IS copies (Fig. 4B) and overall IS density (Fig. S9B)—we also verified that the same exponential distribution of cluster size holds across various phylogenetically distant species (Fig. S10). In chromosomes with both >120 ISs and high IS density, the distribution deviates from an exponential, becoming heavier-tailed (Fig. S9C).

Such a “universal” (exponential) law [46] in chromosomes containing fewer than 120 ISs further supports the existence of a generic stochastic process driving the chromosomal location of ISs, independent of their features. To characterize this process, we considered three models of random locations of ISs along the chromosome, each representing a different hypothesis regarding the accessibility of genomic regions by the ISs: (i) the entire chromosome is accessible to ISs, (ii) accessible regions are restricted to a fraction of the chromosome, and (iii) the number of accessible sites increases proportionally with the total number of ISs—details of the models and their predictions are provided in Supp. Note 1. We find that the model (iii) with proportional accessible sites, and only this model (Supp. Note 1), leads to an exponential distribution that is independent of the number of ISs in the genome. Fitting the data yields an estimated number of accessible sites equal to *∼ 5.4* times the number of ISs per chromosome (Supp. Note 1). Consistently, only this model can account for the observed dependence of the mean cluster size per chromosome as a function of IS counts, up to 120 ISs (Fig. 4C). Beyond this threshold, data are well explained by assuming, instead, that the number of accessible sites saturates (*i.e.* scenario II) at the value predicted for 120 ISs under scenario III, *i.e.*  $\sim 5.4 \times 120 \simeq 648$ IS-equivalent sites (Fig. 4C, Supp. Note 1). More precisely, the best-fitting model is one in which the size of the accessible region is proportional to chromosome size (see Supp. Note 1).

Altogether, these results suggest that cluster formation primarily arises from limited chromosome accessibility, and that the size of the niche (the number of accessible sites) is proportional to the number of ISs.

### Regions of genomic plasticity constitute the primary reservoir for IS niches

**Figure 5.**
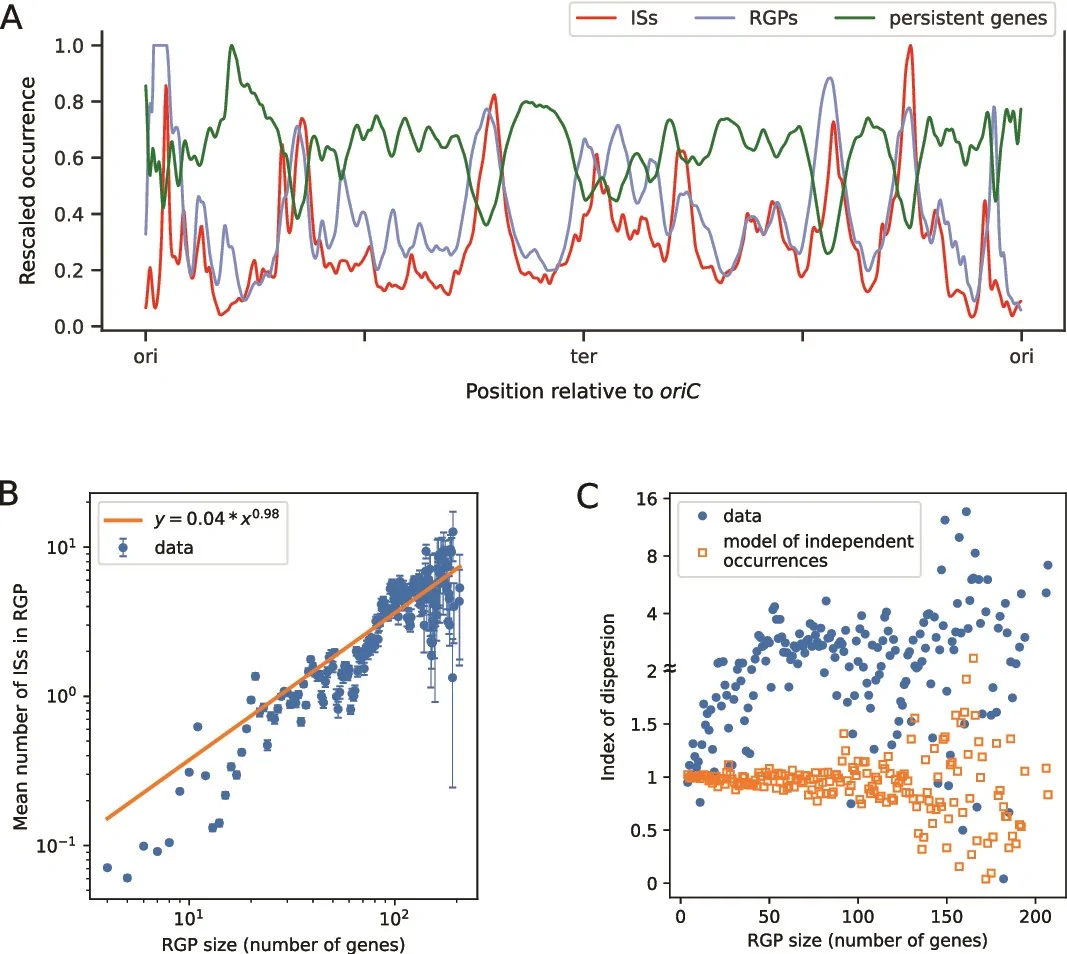
ISs versus RGPs in *E. coli* (see Fig S11 for other species). A) Distribution of unique IS positions (red), persistent gene positions (green) and frequency at which a region is an RGP (blue) across *E. coli* chromosomes. For each profile, values were rescaled to the maximum value within that profile. B) Mean number of IS copies as a function of RGP size (number of genes within each RGP), computed from all RGPs found in *E. coli* chromosomes, using a log–log scale—error bars represent the standard error of the mean. The orange line indicates the best power-law fit, revealing an almost linear relationship. C) Index of dispersion (*i.e.* ratio of the variance to the mean) for the IS content of RGPs as a function of RGP size. The orange symbols represent values obtained in a model in which, within a given RGP, genes have a fixed probability of being an IS, as determined by the fit in (B) (*∼ 1/28* here), independent of other IS occurrences. Note that the *y*-axis uses a semilogarithmic scale to accommodate points with very large values, with a linear scale up to 2 (indicated by the *≈*) and a logarithmic scale above.

To further characterize IS niches, we compared the distribution of ISs along chromosomes with the distribution of conserved versus variable genomic regions. To this end, for several species, we first aligned, normalized, and segmented the chromosomes of all their genomes into 1000 bins. We then computed the occurrence profiles of ISs, persistent (or core) genes, and RGPs [30], the latter only containing accessory genes. In *E. coli*, we observe a strong correlation of IS locations with RGPs, along with a negative correlation with persistent genes (Fig. 5A, see Fig. S11AC for species with significantly lower and higher GC content). More precisely, 66% of ISs are located within RGPs, a proportion that reaches 87% when considering only clustered ISs. The average number of ISs in RGPs is found to scale with RGP size, often in a linear manner (Fig. 5B and Fig. S11BE)—for comparison, there exists no clear relationship between the number of ISs and the size of chromosomes [9, 47] (Fig. S12).

Altogether, these results suggest that RGPs serve as the primary reservoir for IS niches, and that ISs often contribute, in average, to a constant proportion of each RGP in a given species.

### IS occurrences in RGPs are not independent from each other

We investigated whether ISs behave independently within RGPs or if there is evidence of interactions suggestive—from an ecological perspective—of group behavior. To address this, we measured the dispersion of IS copy numbers within RGPs by calculating the variance-to-mean ratio, a metric known as the index of dispersion in ecological studies. Under the assumption of independent IS insertions, this index is expected to be close to *1*, regardless of RGP size—reflecting a Poisson distribution where the probability of an RGP gene being an IS is fixed. However, using the same dataset as that we used to compute the mean number of IS copies for all RGPs of a given size (see Fig. 5B), our results reveal overdispersion, with most index values exceeding *1* (Fig. 5C, Fig. S11CF). We therefore conclude that IS occurrences within RGPs are not independent of each other, but rather indicative of group behavior.

### IS niches are AT-enriched and extend over tens of kb of coding and noncoding DNA

**Figure 6.**
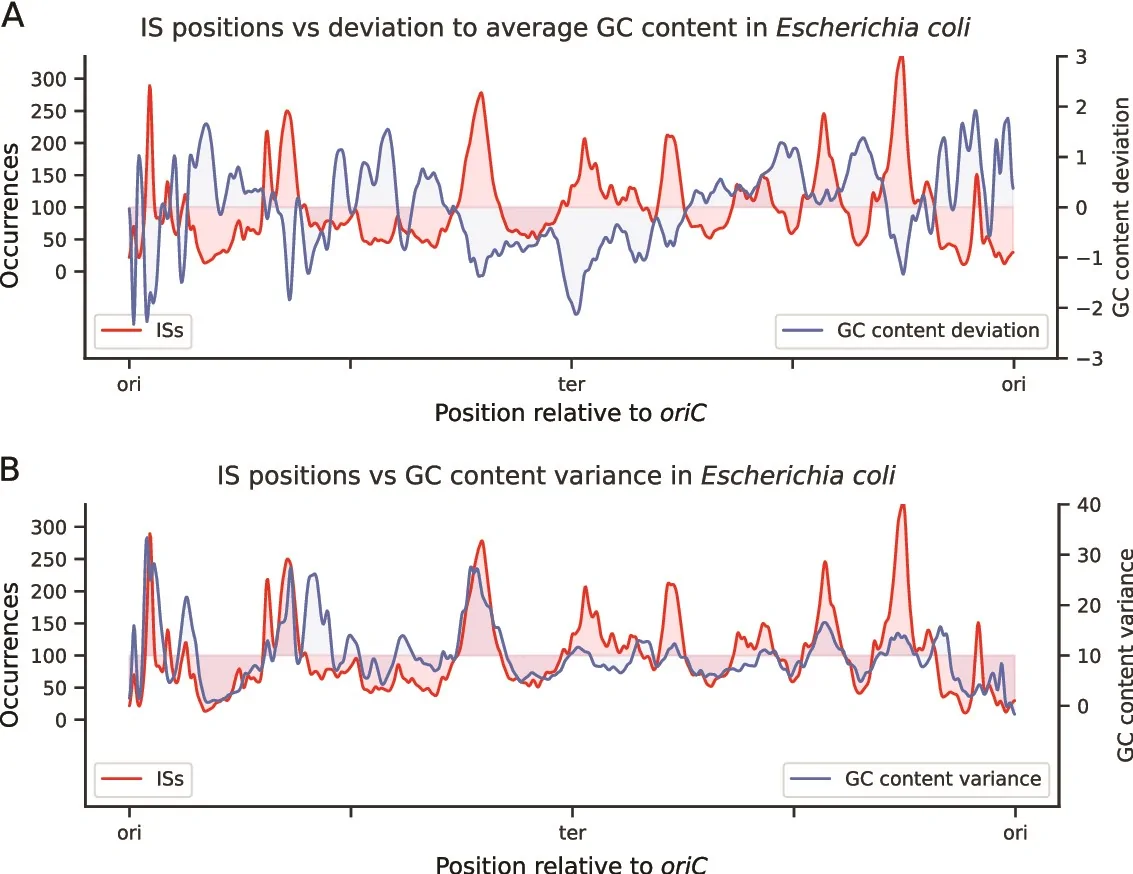
Characterization of the GC content of IS niches in *E. coli* chromosomes (see Fig S13 for other species). A) Distribution of unique IS positions (red) and deviation to average GC content (blue) across *E. coli* chromosomes. Pearson correlation: −0.47. B) Comparison of ISs distribution to the variance in GC content across *E. coli* chromosomes. Pearson correlation: 0.51.

To further investigate the genomic properties of IS ecological niches, we compared the chromosomal distribution of ISs with that of GC content. To this end, we followed the same procedure we used to compute the occurrences of RGPs and persistent genes (see above). Considering three distantly related bacterial species, we find that the IS distribution strongly correlates with the average AT content profile computed over the chromosomes, regardless of the overall GC content of the species (Fig. 6A and Fig. S13AC). This correlation is barely visible in individual genomes but becomes clear when analyzing multiple chromosomes (typically 10 or more in *E. coli*) (Fig. S14), *i.e.* when averaging the signal over evolutionary time. In line with this, and with the preferred location of ISs within RGPs, we also find that the IS distribution strongly correlates, in a given species, with the profile of GC variance computed over its chromosomes (Fig. 6B and Fig. S13BD).

**Figure 7.**
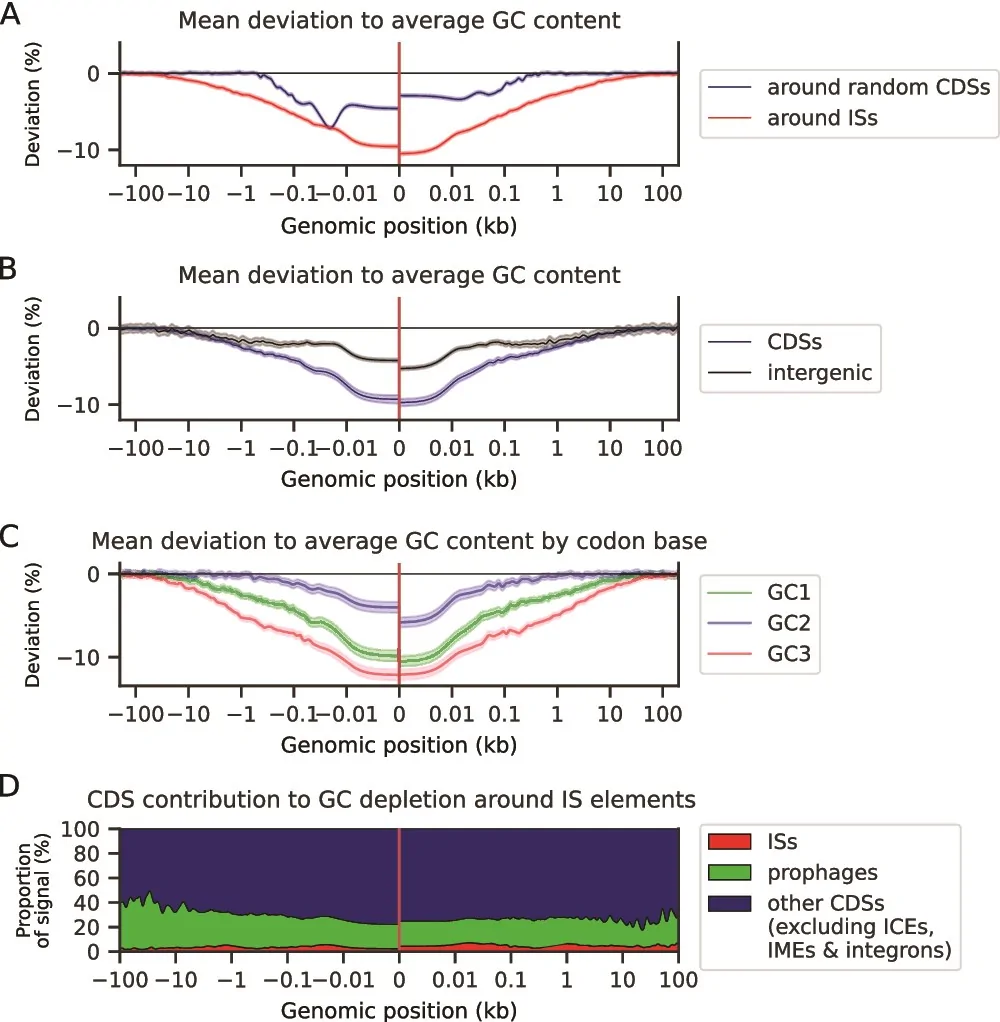
GC content deviation around ISs computed from all 30 409 genomes. A) Relative deviation (in %) from average GC content at a given distance from a CDS (blue) or an IS (red), considering its transcriptional orientation as in Fig. 3. B) Relative deviation (in %) from average GC content at a given distance from an IS, in CDSs (blue) or intergenic sequences (gray). C) Relative deviations from average GC content, shown separately for each codon position. D) Contribution of different types of CDSs (IS CDSs, prophage CDSs, ICE & IME CDSs, integron CDSs and other CDSs) to the GC depletion around ISs. The contribution of integrons, ICEs and IMEs is too small to be visible. Panels A, B, C: the shaded areas (barely visible in A) show the standard errors of the mean.

To assess the generality of this AT enrichment in the vicinity of ISs—and given the impossibility of globally aligning all bacterial genomes—we aggregated all DNA sequences flanking ISs (accounting for IS reading direction) from our dataset of 30 499 genomes, and computed the average GC content across the corresponding pile-ups. Figure 7A presents the resulting profile as a function of the distance from the IS borders and compares it with profiles generated by aggregating sequences around random CDSs. We find a marked GC depletion (*i.e.* AT enrichment) around ISs that extends over tens of kilobases. Such AT enrichment can stem from two main sources: either a depletion of CDSs (which are richer in GC compared with intergenic regions), or an intrinsic AT enrichment occurring in CDSs, intergenic regions, or both. By quantifying contributions to the signal (Supp. Note 2), we find that the observed trend arises primarily from intrinsic AT enrichment (Fig. S15), which concerns both CDSs and intergenic regions (Fig. 7B). Moreover, it affects CDSs independently of the codon position (Fig. 7C), and regardless of the GC content of genomes. The strongest effect, however, systematically concerns the codon position with the highest overall GC content (Fig. S16).

Regardless of a genome’s overall GC content, ISs are thus found in chromosomal regions that accumulate more AT-rich sequences than the rest of the genome. This AT enrichment affects both coding—independently of codon position—and noncoding sequences, and it extends over regions up to *∼*100 kb around ISs.

### AT enrichment primarily originates outside known MGEs and affects them

Given that plastic regions of genomes are characterized by frequent HGT [12, 30], and that the GC content of horizontally acquired genes tends to differ significantly from that of the host genome [48] (Fig. S17), we investigated the relative contribution of known MGEs to the AT enrichment of CDSs around ISs, across all prokaryotic genomes. To this end, following methods in [12], we considered four main categories: ISs, prophages, identifiable genes of ICEs/IMEs and integrons (Methods). We find that *∼*60% of the AT enrichment of CDSs around ISs arises from genes other than known MGEs (Fig. 7E). Indeed, the main MGE contribution comes from prophages and accounts only for *∼*30%, whereas ISs account for <5%, and ICEs and integrons together for <0.5%. Moreover, prophage sequences tend to become increasingly AT-rich the closer they are to an IS (Fig. S18A), a trend not observed for ISs and integrons, and only weakly for ICEs/IMEs (Fig. S18B). The majority of the AT-enrichment signal around ISs is therefore not attributable to known MGEs. Moreover, some MGEs, although already more AT-rich than the rest of the genome, exhibit an even stronger AT-enrichment when located near ISs.

### Ecological niche differentiation of MGEs

**Figure 8.**
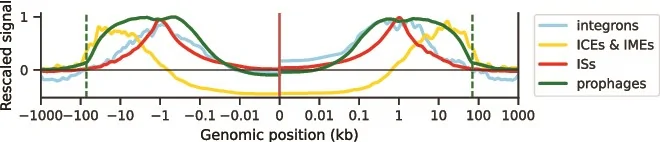
Distribution of MGEs around ISs. The signal was rescaled such that for a given MGE *M*, we compute $[f(M) - f_{base}(M)]/[f_{max}(M) - f_{base}(F)]$, with *f(M)* the fraction of sequences for which this position corresponds to the MGE, $f_{base}(M)$ the average fraction in the genome, and $f_{max}(M)$ the maximum value reached by *f(M)*. The vertical dashed lines indicate a characteristic length of 70 kb associated with a sharp change in the distribution of prophage locations.

An analysis of the genomic distribution of MGEs around ISs (Fig. 8) further reveals that the identifiable genes of ICEs/IMEs (Methods) tend to locate farther from ISs than prophages or integrons. Moreover, these ICE/IME genes are depleted in the immediate vicinity of ISs and they are, on average, more GC-rich than other MGEs (Fig. S19). A characteristic length of *∼*70 kb associated with prophage locations is also clearly visible (Fig. 8, in green). A similar length has been previously reported and interpreted as an upper limit for chromosomal integration of prophage segments [49, 50], which are subsequently subject to genetic degradation [49]. This suggests that prophages close to ISs may primarily result from recent HGT.

Altogether, these results reveal a partitioning of the genomic space occupied by MGEs around ISs, which we interpret as evidence of ecological niche differentiation among MGEs.

## Discussion

We conducted a comprehensive ecological analysis of ISs both within and across genomes. In doing so, while keeping in mind the limitations of automated detection methods and the lack of information on the evolutionary trajectories of ISs and their genomic context (Supp. Note 3), we uncovered several phenomena that are difficult to interpret outside the framework of ecology. In particular, we found that although genomes generally provide sufficient resources for ISs to coexist, RGPs, which are the most variable chromosomal regions across strains of a given species, constitute the main reservoir for IS niches. We also found that regardless of the species studied—including species with very different GC content—the size of the IS ecological niche varies linearly with the number of ISs, always maintaining the same average proportion: for each additional IS in a chromosome, there are *∼ 5.4* additional niche sites. These findings have two key implications. First, the observation of the same property across most species suggests the existence of very generic—poorly understood—mechanisms, reminiscent of the universal mechanisms underlying the scaling laws observed in the functional composition of bacterial genomes [51, 52]. Second, the question of causality arises: Do ISs drive the expansion of their own niches—or of the RGPs—or do larger RGPs provide more space for ISs? The first scenario would correspond to a “niche construction” mechanism, where the insertion of an IS in a particular location increases the likelihood of subsequent IS insertions or promotes the extension of RGPs—for instance through large IS-mediated duplications of chromosomal segments, the insertion of composite transposons and mobilizable units, or the integration of IS-carrying plasmids. The second scenario would still leave open the question of what determines the frequent observation of a constant fraction of ISs within RGPs. Distinguishing these scenarios will certainly require a high amount of high-resolution temporal evolutionary data. In all cases, we find that IS occurrences within RGPs do not behave independently of each other, which could be because the presence of one IS may increase the likelihood of others inserting nearby. Rather than acting as isolated, selfish elements—a common explanation for the spread of TEs among organisms [53],—we thus propose that ISs exhibit group behavior, a hallmark of collective phenomena in microbial ecology (of organisms).

Perhaps unsurprisingly, in all the species we investigated in detail—and for which a large number of genomes were available—IS niches are found within the most dynamic chromosomal regions, the RGPs, where HGT is particularly frequent. Nevertheless, the observation of a specific—often linear—scaling of IS number with RGP size suggests that the internal dynamics of these RGPs is driven by specific composition rules. In this context, knowing that RGPs can contain hundreds of genes, and that IS-surrounding genomic patterns—such as the GC depletion signal or the location of MGEs—extend up to 100 kb, the HGT hotspots identified in previous studies (which typically comprise only a few percent of the genome [12]) may reflect finer-scale structuring of ISs and MGEs within these RGPs. Along this line, our observation of MGEs and ISs occupying specific genomic locations relative to each other, with in particular identifiable ICE/IME genes often located farther from ISs than prophages, suggests the existence of still poorly understood mechanisms specifying the spatial organization of the ecological niches of ISs and, more generally, of MGEs. The widespread presence of terminator-like sequences upstream of IS CDSs, which are found both within the IS itself and just outside it, also suggests the existence of regulatory mechanisms mitigating IS transposase expression. This regulation could prevent excessive transposition activity, which might otherwise lead to an explosion of IS copies detrimental to the host genome [3], and, hence, contribute to the survival of ISs within genomes. It is important to emphasize that the observed genomic patterns ultimately result from selective processes. This is particularly evident in the fundamental genomic properties associated with IS niches. Specifically, the observed bias of IS niches toward intergenic regions and areas that do not disrupt the functional organization of genomes supports a major role of purifying selection in shaping IS distributions. Nevertheless, our finding that ISs more frequently disrupt genes involved in defense mechanisms, DNA repair and extracellular structures also suggests that some events may involve positive selection. It is also possible that these contexts favor the survival of the ISs themselves.

The specific depletion of GC content in the vicinity of ISs—strongest near the IS and extending up to 100 kb—introduces a new dimension to the discussion on the origins of the heterogeneity of the GC content across bacterial genomes [54] and within them. Namely, a commonly proposed explanation for intra-genome heterogeneity is that genes with higher recombination rates tend to have higher GC content [55, 56], and that recombination is often more frequent in core genes than in accessory genes (see e.g. [57–59]). Yet, the underlying reason why recombination is GC-biased remains actively debated—see e.g. [60]. Some have argued for a mechanistic bias inherent to the recombination process itself [55], whereas others invoke a selective pressure favoring optimal expression of conserved genes [56, 60–62]. Here, we first note that HGT-associated hotspots have been shown to harbor higher recombination rates [12]. Under a purely mechanistic hypothesis, one would thus expect an enrichment of GC content, at least in the intergenic regions near ISs, given that many of these ISs are found within such hotspots [12]. However, we observe the opposite: a pronounced GC depletion, for both noncoding flanking regions and CDSs, and irrespective of codon position for the latter. This contradiction therefore supports the alternative explanation of a relaxed selective pressure within IS-associated niches. Yet, it remains unclear how this could also account for the AT enrichment specifically observed in intergenic regions. One possibility is that part of the signal arises from an enrichment of AT-rich subsequences that are prone to gene silencing and involved in the regulation of horizontally acquired genes [63], given that xenogeneic silencing has been shown to both act as a selective force shaping the GC content of genomes [64] and to influence the ecological niches of ISs [35].

Altogether, our findings point to a situation involving both physical and ecological isolation of ISs, and more broadly of MGEs. Physical isolation refers to the tendency of these elements to localize in specific regions of the host genome, whereas ecological isolation, also called isolation by environment [65, 66], would reflect selective pressures in these regions that differ from those acting on the rest of the genome.

Beyond the niche characteristics shared by all IS families, our results also reveal that some families exhibit distinct niche specificities with respect to flanking gene synteny, distance to neighboring genes, or the upstream presence of transcription terminators. Because some of these specificities are expected to depend on the tool used to identify ISs, we have verified that the main findings we report remain robust when switching between annotation tools (see Supp. Note 3). In this context, IS-specific properties are likely linked to family-specific target sequence preferences, as well as to specific expression properties and functional roles. This is further supported by the consistency between some of the patterns identified in our large-scale study and behaviors reported in more specific studies. For instance, members of the IS*30* family—one of the few families that insert between syntenic gene pairs more frequently than expected—have been reported to activate neighboring genes by creating hybrid promoters [10], which might ultimately confer a benefit to the host. Another example is the IS*481* family, in which many elements are found juxtaposed to flanking CDSs: a similar bias was recently reported in *Bordetella pertussis* and shown to be associated with the regulation of gene expression in the flanked gene [43]. Finally, our identification of terminator-like sequences both downstream of non-IS CDSs and upstream of specific IS families strongly suggests that these sequences indeed function as transcription terminators potentially attenuating the expression of the IS transposase, as demonstrated in a previous work on the IS*200*/IS*605* family [44]. Demonstrating the functional impact of all identified patterns in a systematic manner may nevertheless prove challenging, as it would require both a refined case-by-case analysis of genomic sequences and experimental testing of the resulting predictions.

From a broader perspective, by framing IS biology within a quantitative ecology of transposons, we anticipate new lines of investigation into their evolutionary dynamics, with new types of questions such as: To what extent do ISs compete with one another? Can ISs specifically collaborate, either with each other or with other MGEs? Is there a process akin to ecological succession when IS elements and other MGEs colonize new genomic regions? Pursuing these directions will not only clarify the roles of ISs and MGEs in genome evolution but also help establish a unifying ecological framework for understanding the mobile genome.

## Supplementary Material

Supplementary_material_wrag159

## Data Availability

A repository containing the scripts used to generate the results of the article can be found at https://zenodo.org/records/20623700. The public data used in this study are not shared, as the corresponding compressed files are too large (several hundreds of GB). The information required to access these data is provided in the Methods section of the article.
