# Supplementary material for: Characterizing the ecological niche of insertion sequences within prokaryotic genomes
Source: ISME J. 2026 Jul 11;20(1):wrag159. doi: 10.1093/ismejo/wrag159 (PMC13548323; doi:10.1093/ismejo/wrag159)
Supplement: Supplementary file 1 [file supplementary_material_wrag159.zip › supp_mat_wrag159.pdf]

Supplementary Information – Characterizing the ecological niche of insertion sequences within prokaryotic genomes

Flora Gaudillière-Jami [1,✉], **Sophie Abby** [1], **Thomas Hindré** [1], **and Ivan Junier** [1,2,✉]

[1]TIMC, UMR 5525, VetAgro Sup, Grenoble INP, CNRS, Université Grenoble Alpes, Grenoble, France; [2]Centre de Biologie Intégrative (CBI), Université de Toulouse, CNRS, UPS, 31000 Toulouse, France

1 Random location models for the formation of IS clusters **1**

2 Quantifying the contribution of features to the GC depletion signal **3**

3 Caveats and limitations **5**

4 Supplementary figures **7**

5 Supplementary tables **23**

Supplementary Note 1: Random location models for the formation of IS clusters

We test whether the clustering properties of ISs can be explained by models in which their genomic locations are randomly assigned under three different scenarios: (I) a model where the entire chromosome is accessible (according to a uniform probability distribution), (II) a model where IS-accessible regions are limited to a fraction of the chromosome, and (III) a model where the number of accessible sites scales linearly with the number of ISs.

General framework

Let us first consider a chromosome containing n ISs plus A accessible sites with which ISs can be exchanged, i.e. the permutation of their location does not alter the selective pressure acting on the host. Considering ISs as occupied sites, this means that, for a given configuration of IS locations, there are n occupied and A empty accessible sites (see panel A of the associated figure). It also means that any permutation of the IS positions among these accessible sites corresponds to a valid configuration that can be sampled during the course of evolution. One can then ask: what is the probability $P_{n,A}(k)$ that a given cluster has size k (including the case $k = 1$), assuming an equiprobable distribution over all possible configuration of IS locations?

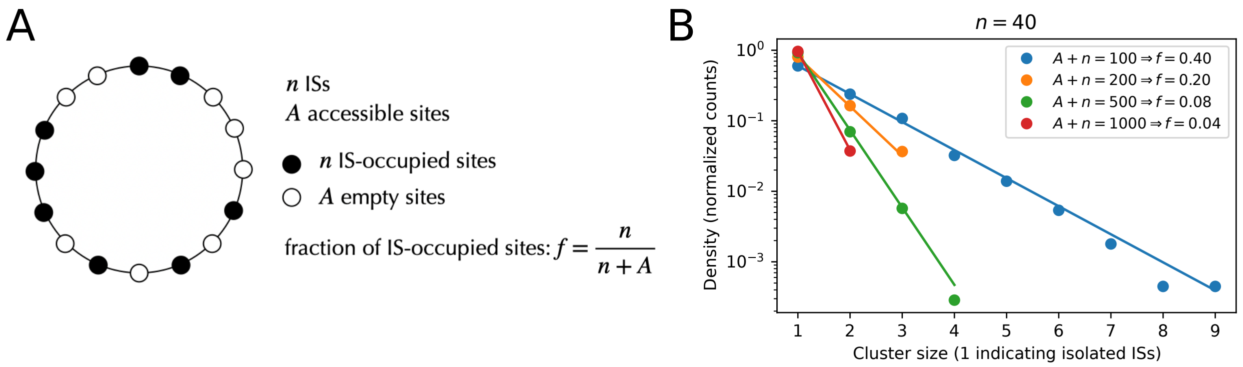

A. **Scheme of the random location models.** B. **Comparison between simulations (points) and analytical predictions (lines) given by Eq. S1.**

$P_{n,A}(k)$ can be computed by noting that it corresponds to the probability that an occupied site, flanked on its left by an empty site, is followed on its right by $k-1$ occupied sites and an empty site at the kth position. Defining $f_{n,A} = \frac{n}{n+A}$ as the fraction of accessible sites that are occupied, we have:

$$P_{n,A}(k) = (1 - f_{n,A}) {f_{n,A}}^{k-1}, \quad \textbf{(S1)}$$

a formula that can be verified by simulating the (evolutionary) sampling of all possible configuration of IS locations (see panel B of the associated figure).
The corresponding mean size of the clusters, $m_A(n)$, as a function of n is given gy:

$$m_A(n) = \frac{\sum_{k=2}^{\infty} k P_{n,A}(k)}{\sum_{k=2}^{\infty} P_{n,A}(k)} = \frac{\sum_{k=2}^{\infty} k {f_{n,A}}^{k-1}}{\sum_{k=2}^{\infty} {f_{n,A}}^{k-1}}. \quad \textbf{(S2)}$$

Dropping the subindexes of $f_{n,A}$ for lightness, we obtain:

$$m_A(n) = \frac{n f^n - (n+1) f^{n-1} + 2 - f}{(1-f)(1-f^{n-1})} \quad \textbf{(S3)}$$

In particular, for values of n such that $n f^{n-2} \ll 1$, which occurs typically at $n \simeq 10$ in the case of the fit to the genomic data (see Fig. 4C), one has:

$$\begin{aligned} m_A(n) &\simeq \frac{2-f}{1-f} && \textbf{(S4)} \\ &= 2 + \frac{n}{A} && \textbf{(S5)} \end{aligned}$$

Scenario I: full access to the chromosome

In this scenario, all ISs can be located at any position along the chromosome. In the terminology of our above general model, this means that IS locations can be exchanged with any other gene, such that $n + A$ represents the chromosome length (in number of genes). For simplicity, in Fig. 4C, we used $A = 4000$, which corresponds to the typical length of the chromosomes analyzed. Nevertheless, one can compute the mean cluster size $m(n)$ as a function of n by considering the specific length of each chromosome. In this case, calling N_C the total number of genes (excluding ISs) and n_C the number of ISs in a chromosome C, one has:

$$m(n) = \frac{1}{\Omega_n} \times \sum_{\{C | n_C = n\}} m_{A=N_C}(n), \quad \textbf{(S6)}$$

where $\{C \mid n_C = n\}$ denotes the set of chromosomes C for which $n_C = n$, $\Omega_n = |\{C \mid n_C = n\}|$ is the number of such chromosomes, and $m_{A=N_C}(n)$ is given by Eq. S3.
Plots then show that the two approaches yield very similar results (blue curves in the associated figure).

Scenario II: limited access to the chromosome

In this scenario, only some part of the genome can be accessed by ISs. In the terminology of our general model, this means that A represents some part of the genome. One can then consider two distinct situations: i) A is a fixed quantity, independent of the genome length; ii) A is a fraction of the total genome. For simplicity, in Fig. 4C, we used a fixed quantity $A = 5.4 \times 120$ as the maximum size of the accessible genomes coming from scenario III (see below). Nevertheless, part of the large variation of data for $n \geq 120$ can be explained considering Eq. S6 with A that is a fixed part of the genome, i.e. using $A = \alpha N_C$ such that:

$$m(n) = \frac{1}{\Omega_n} \times \sum_{\{C | n_C = n\}} m_{A=\alpha N_C}(n). \quad \textbf{(S7)}$$

In particular, using $\alpha = 0.16$ provides a reasonable fit of the data (orange curve in the associated figure).

Scenario III: the size of IS niche scales linearly with the number of ISs

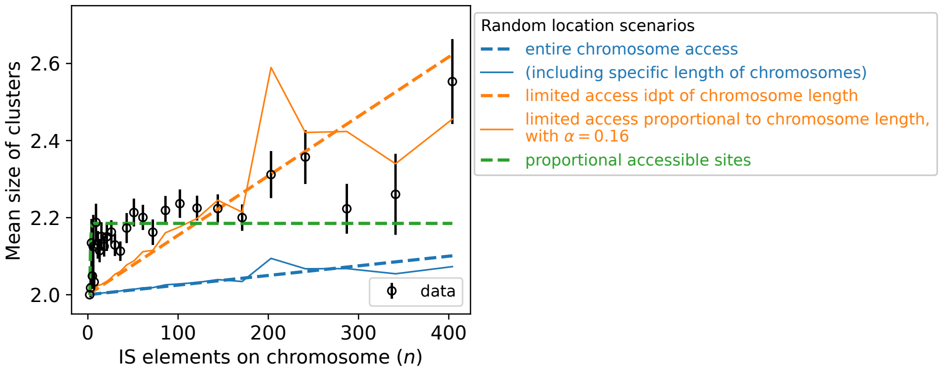

Effect of considering the specific length of each chromosome in models (solid curves) compared to considering a single average value (dashed curves). The model of proportional accessible sites is not concerned since the parameter of the model is independent of the length of the chromosome.

In this scenario, the additional presence of an IS is accompanied by new genes with which ISs can be exchanged. In the simplest case, we can assume that the number of new accessible sites is proportional to the number of additional ISs such that the size of the IS niche scales with the number of ISs, i.e., $A = \beta n$ with $\beta \geq 0$. In this case, one has:

$$f_{n,A} = \frac{n}{n+A} = (1+\beta)^{-1} \quad \textbf{(S8)}$$

which is independent of both n and the size of genomes. Note, then, that in this case, the size distribution of clusters follows an exponential distribution that is independent of these factors as well, just a observed in experimental data (see Fig. 4B).
Regarding the mean size of clusters, we obtain:

$$m(n) = \frac{n(1+\beta)^{-n} - (n+1)(1+\beta)^{-(n-1)} + 2 - (1+\beta)^{-1}}{(1-(1+\beta)^{-1})(1-(1+\beta)^{-(n-1)})} \quad \textbf{(S9)}$$

with, in the limit of large n:

$$m(n) \simeq 2 + 1/\beta. \quad \textbf{(S10)}$$

This scenario, with $\beta \simeq 5.4$, explains particularly well the data for $n \leq 120$ (green dashed curve in the associated figure; see also Fig. 4C).

Supplementary Note 2: Quantifying the contribution of features to the GC depletion signal

To quantitatively assess the contribution of CDS and intergenic regions to the GC depletion signal, we generated GC content profiles separately for CDS and intergenic positions, using NCBI metadata available for each genome. In this context, as demonstrated below, the contribution of a feature-specific signal at position x reads:

$$\lambda_F[x] = (q_F[x] - q_{F,\infty})\frac{GC_{F,\infty} - GC_\infty}{GC_\infty} + q_F[x]\frac{GC_F[x] - GC_{F,\infty}}{GC_\infty} \quad \textbf{(S11)}$$

where, for a given feature F (either CDS or intergenic): $GC_F[x]$ is the corresponding GC content profile, $GC_{F,\infty}$ is the corresponding average GC content at positions far from the IS (in practice, at a distance of 1 Mb), $q_F[x]$ is the fraction of times F is found at position x, and $q_{F,\infty}$ is the fraction of times F is found at positions far from the IS. GC_∞ is the overall, i.e. feature-independent, average GC content at positions far from the IS.
This contribution consists of two terms that can be analyzed separately. The first term, which equals 0 if $q_F[x] = q_{F,\infty}$, reflects either an under-representation ($q_F[x] < q_{F,\infty}$, as observed for CDS) or an over-representation ($q_F[x] > q_{F,\infty}$, as observed for intergenic regions) of the feature F. The second term, which equals 0 if $GC_F[x] = GC_{F,\infty}$, reflects an AT-enrichment if $GC_F[x] < GC_{F,\infty}$, which is observed for both CDSs and intergenic regions. In the panels B and C of Fig. S15, we therefore computed the relative contribution of each of these terms to $\lambda_{\text{CDS}}[x]$ and $\lambda_{\text{intergenic}}[x]$, respectively, up to 10 kb, where the GC depletion signal remains clearly visible (see Fig. 7A).

Application to the depletion signal around ISs

For Fig. S15A, we applied Eq. S11 using the set of features $\mathcal{F} = \{\text{intergenic}, \text{CDS}\}$. Note that for both intergenic and CDS features, the two terms in Eq. S11 are negative, allowing to assess the relative contribution of each term, as done in Fig. S15BC. It is then interesting to note that the first term is negative for different reasons: CDSs tend to be both under-represented ($q_{\text{CDS}}[x] < q_{\text{CDS},\infty}$) and more GC-rich ($GC_{\text{CDS},\infty} > GC_\infty$), whereas intergenic regions tend to be over-represented ($q_{\text{intergenic}}[x] > q_{\text{intergenic},\infty}$) and more AT-rich ($GC_{\text{intergenic},\infty} < GC_\infty$). By contrast, the second term is negative for both CDSs and intergenic regions due to their AT-enrichment, i.e., $GC_F[x] < GC_{F,\infty}$ for $F \in \{\text{intergenic}, \text{CDS}\}$.
For Fig. 7D, we quantify the contribution of known MGEs by applying Eq. S11 in the context of CDSs only, separating the CDS of MGEs from the rest. The set of features in this context is therefore $\mathcal{F} = \{\text{CDS(IS)}, \text{CDS(phage)}, \text{CDS(ICE/IME)}, \text{CDS(integron)}, \text{CDS(other)}\}$.

Demonstration of Eq. S11

We consider a complete set of disjoint features $\mathcal{F}$ such that, denoting by $q_F[x]$ the probability of finding a feature F at a genomic position x, we have:

$$\sum_{F \in \mathcal{F}} q_F[x] = 1 \qquad \textbf{(S12)}$$

Now, what is the GC content at position x? Or, equivalently, what is the probability, denoted hereafter $GC[x]$, of finding a GC base pair? By definition, we have:

$$GC[x] = \sum_{F \in \mathcal{F}} q_F[x] \times GC_F[x] \qquad \textbf{(S13)}$$

where $GC_F[x]$ denotes the GC content associated with feature F, or equivalently, the (conditional) probability of finding a GC base pair given that feature F is present.
Next, let $\bullet_\infty$ indicate the value of the quantity $\bullet$ when $x \to \infty$. $r_{GC}[x]$, the relative GC content at position x with respect to the signal at long distances, is defined as:

$$r_{GC}[x] = \frac{GC[x] - GC_\infty}{GC_\infty} \qquad \textbf{(S14)}$$

which can also be written as:

$$r_{GC}[x] = \sum_{F \in \mathcal{F}} q_F[x] \times \frac{GC_F[x] - GC_\infty}{GC_\infty} \qquad \textbf{(S15)}$$

Note here that GC_∞ is not necessarily equal to the average value of the GC content over the genome. Actually, it is not in the most general case.
Now, using the fact that $r_{GC,\infty} = 0$, Eq. S15 leads to:

$$\sum_{F \in \mathcal{F}} q_{F,\infty} \times \frac{GC_{F,\infty} - GC_\infty}{GC_\infty} = 0. \qquad \textbf{(S16)}$$

Substracting this equation from Eq. S15, we obtain:

$$r_{GC}[x] = \sum_{F \in \mathcal{F}} \left(q_F[x] \times \frac{GC_F[x] - GC_\infty}{GC_\infty} - q_{F,\infty} \times \frac{GC_{F,\infty} - GC_\infty}{GC_\infty} \right) \qquad \textbf{(S17)}$$

which can be written as:

$$r_{GC}[x] = \sum_{F \in \mathcal{F}} \left((q_F[x] - q_{F,\infty}) \times \frac{GC_{F,\infty} - GC_\infty}{GC_\infty} + q_F[x] \frac{GC_F[x] - GC_{F,\infty}}{GC_\infty} \right) \qquad \textbf{(S18)}$$

As a result, the contribution $\lambda_F[x]$ of the feature F to the relative GC content at position x reads:

$$\lambda_F[x] = (q_F[x] - q_{F,\infty}) \times \frac{GC_{F,\infty} - GC_\infty}{GC_\infty} + q_F[x] \frac{GC_F[x] - GC_{F,\infty}}{GC_\infty}, \qquad \textbf{(S19)}$$

which is Eq. S11.

Supplementary Note 3: Caveats and limitations

Caveats in the analysis of local properties of IS ecological niches

When analyzing the local properties of the ecological niches occupied by ISs within host genomes, it should be kept in mind that, although ISs are typically regarded as elements of recent origin (see e.g. (1)), the observed local context of an IS may not be identical to the ancestral context of insertion, as this context could have changed by subsequent genomic alterations such as deletions, insertions, or rearrangements. Reconstructing the evolutionary trajectories of both ISs and their context is notoriously challenging, if not impossible, and lies beyond the scope of our study. Instead, and despite potential divergence from ancestral states, here we interpret the observed local contexts in which ISs are found as ecological niches in which they are able to survive.

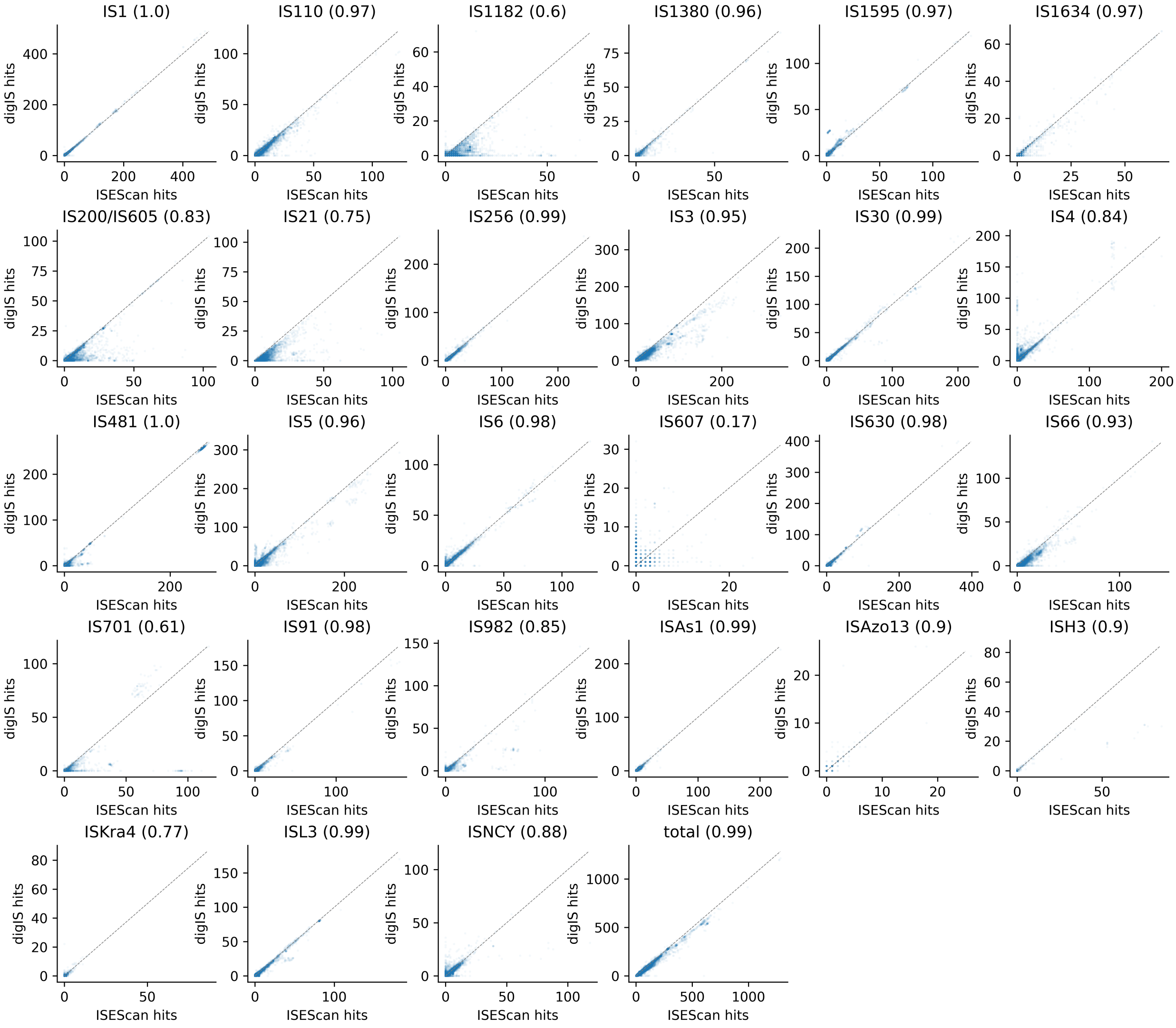

Cross-validation of digIS hits. ISEScan and digIS outputs were compared for a subset of 21,298 genomes. Plots show the number of copies detected in a given genome for each family by either ISEScan (x-axis) or digIS(y-axis). Pearson correlations between the two distributions are indicated between parentheses.

Cross-validation of digIS outputs

A cross-validation was performed by comparing digIS outputs with those of ISEScan (2) for a subset of 21,298 genomes. Overall, detection of IS elements is consistent between the two tools: the number IS copies detected for each family in each genome is highly correlated (see Figure above). In cases where the tools report widely different numbers of copies, ISEScan tends to report more copies than digIS. For IS*1182* and IS*607* families, the two tools report significantly different results (correlation coefficients of only 0.60 and 0.17, respectively). The manual examination of copies reported exclusively by one tool showed that while IS*1182* copies reported by ISEScan only were presumably true positives missed by digIS, IS*607* copies flagged by ISEScan were most likely false positives (no significant similarity with any copy listed on the ISFinder database (3)). Additionally, digIS also detects ISs

belonging to the IS*H6*, IS*Lre2*, and Tn3 families, which is not the case for ISEScan. Because of these findings, we chose to work on digIS outputs: despite the fact that our analysis likely excludes true positives for families other than IS*607*, the significant number of hits reported by digIS was more than sufficient to perform statistically discriminating analyses while avoiding the inclusion of potential false positives.

We also note that some IS-specific properties were not always robust when analyzed ISEScan instead of digIS. In particular, specific peaks in the distances separating ISs from their flanking genes did not always coincide between the two tools. For these properties, the quality of annotation of ISs, as well as the annotation of their limits, plays a crucial role. In the manuscript, we therefore discuss properties and patterns that are observed using either tool.

Limitations and robustness of automated detection approaches

The digIS approach, based on HMMER models, offers greater flexibility than BLAST-based methods and is specifically designed to detect distant IS elements. However, it still relies on known, curated IS sequences from ISfinder, introducing a potential bias towards detecting elements similar to those already characterized. Due to the large size of our dataset, manual curation was not feasible, and despite restricting our analysis to complete IS elements, some false positives may remain. Similarly, the identification of MGEs other than ISs (prophages, ICEs and IMEs, and integrons) was automated using DB-Scan SWA, CONJscan, and IntegronFinder, respectively, without manual verification. As a result, the reported MGE distributions within individual genomes may not be entirely precise.

Nonetheless, our goal is not to analyze specific genomes in detail, but to provide a broad overview of IS distribution patterns within and across genomes. Minor inaccuracies in IS and MGE detection are unlikely to significantly influence our findings, a conclusion supported by the consistency of our results with previous large-scale studies of IS distribution. In other words, the trends we observe are robust and unlikely to be driven by isolated detection errors.

Supplementary Note 4: Supplementary figures

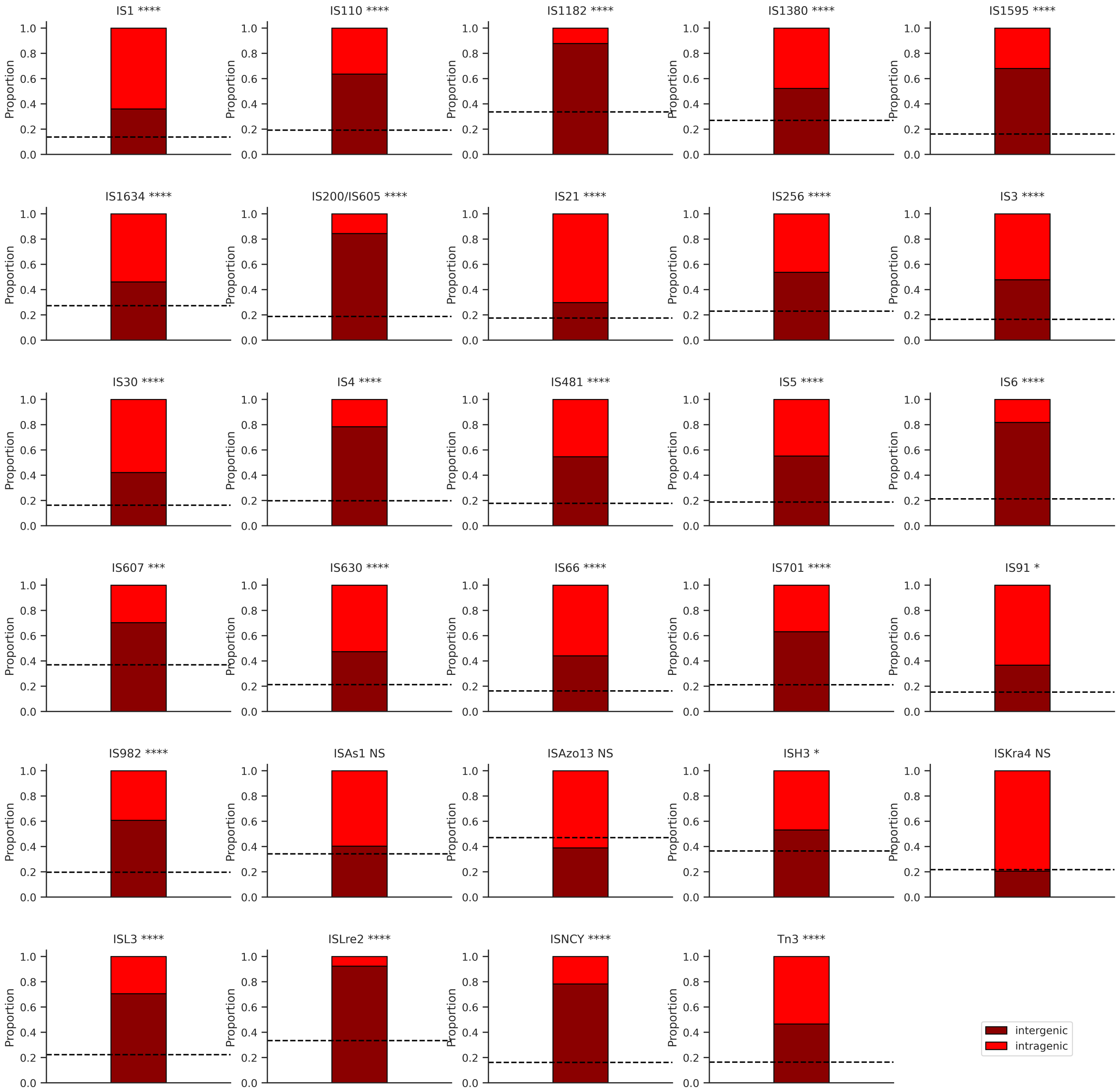

Figure S1. Complementary to Fig. 2A. **Proportion of intergenic ISs for each IS family.** The whole chromosomal proportion indicated by the horizontal dashed line is computed based on the genomes in which the corresponding IS is present. The ISH6 family is not shown as no data was available. Statistical significance was computed using a Chi2 test. NS: *P* value > 0.05. *: *P* value < 0.05. **: *P* value < 0.01. ***: *P* value < 0.001. ****: *P* value < 0.0001.

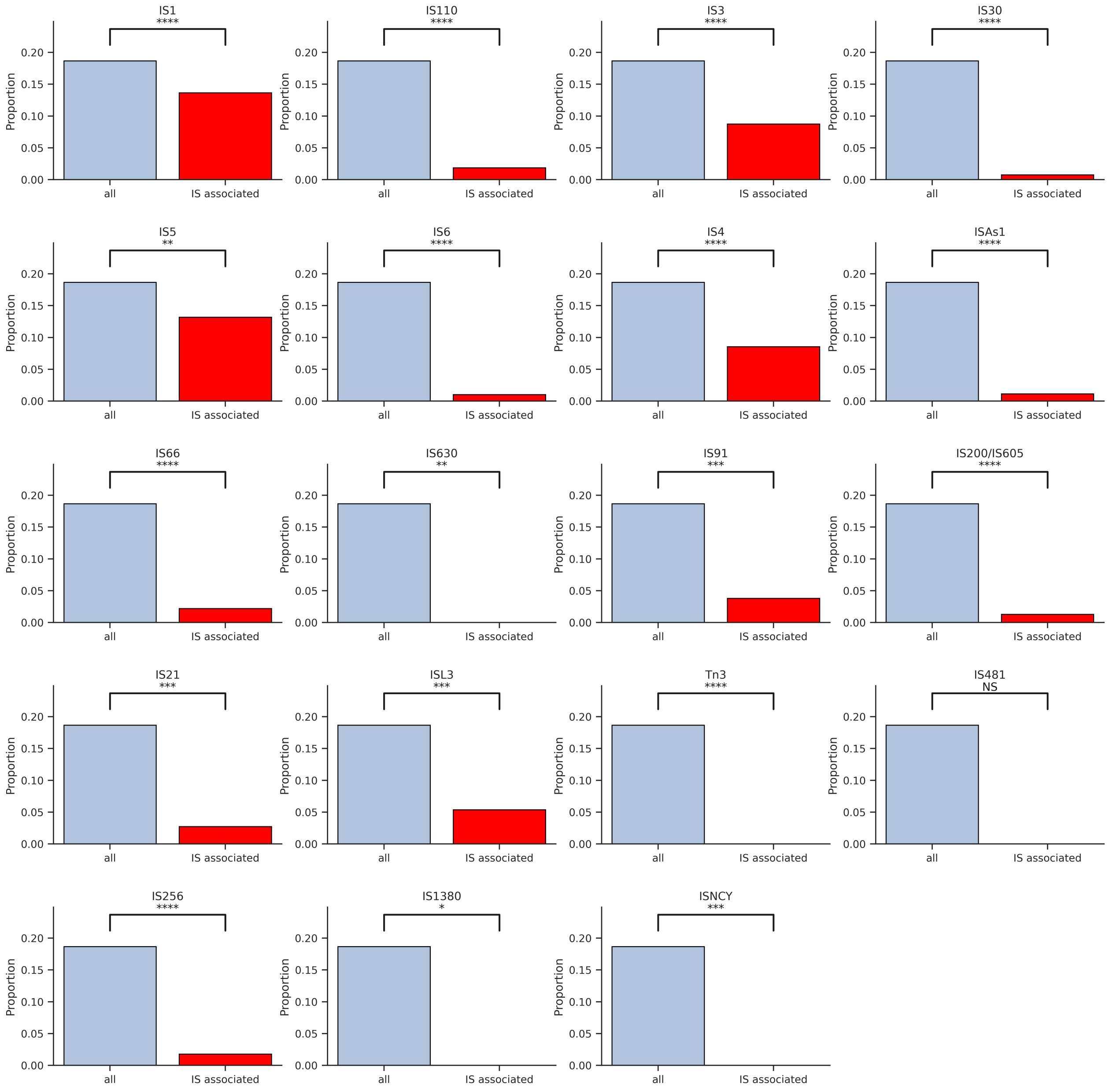

Figure S2. Complementary to Fig. 2B. **In *E. coli* , fraction of intergenic regions found inside of an operon (blue) versus fraction of intergenic IS found inside of an operon (red), shown by IS family.** Statistical significance was computed using a Chi2 test. NS: *P* value > 0.05. *: *P* value < 0.05. **: *P* value < 0.01. ***: *P* value < 0.001. ****: *P* value < 0.0001.

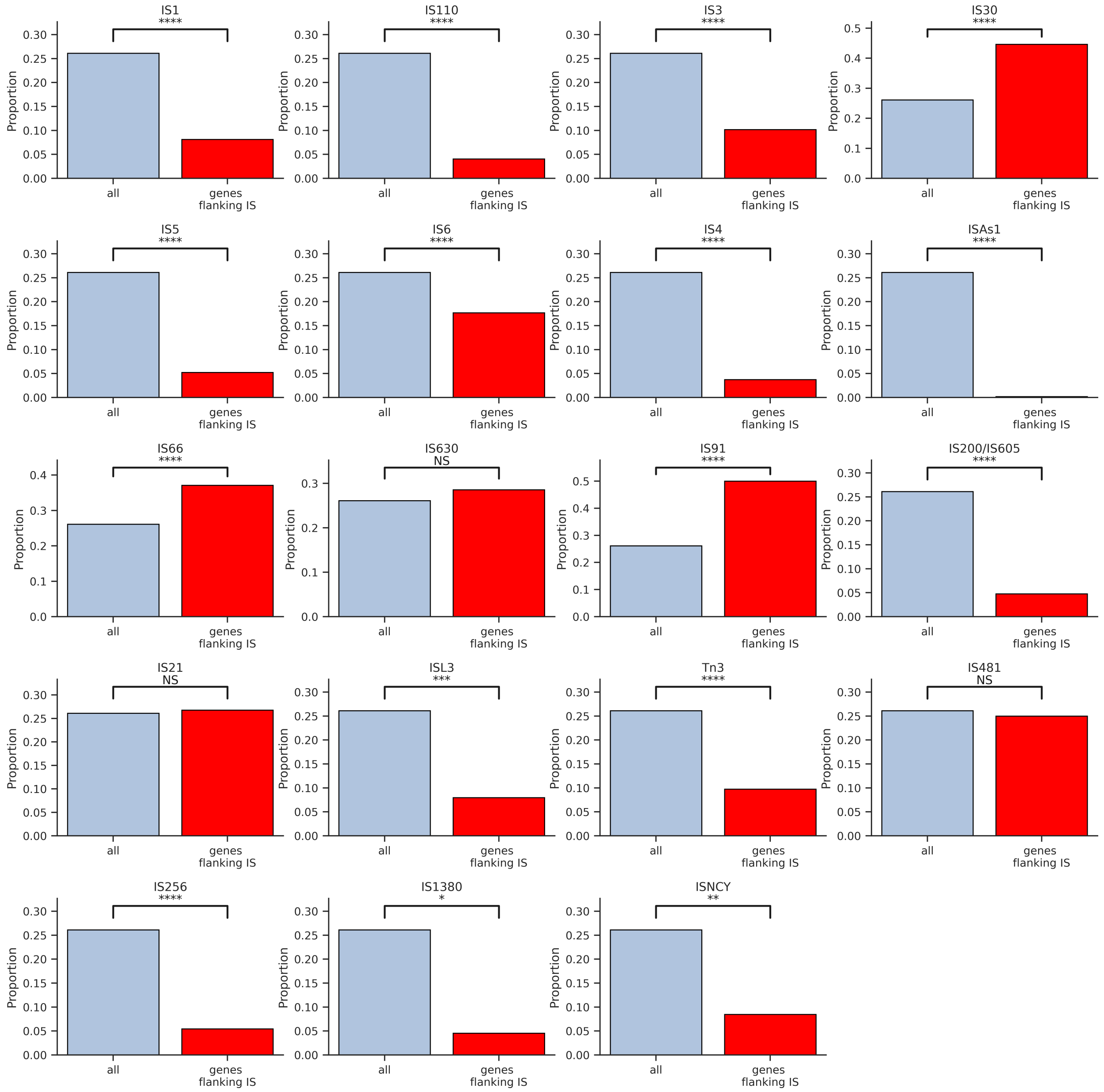

Figure S3. Complementary to Fig. 2C. **Fraction of *E. coli* syntenic neighbor genes among genes that do not belong to the same operon in the genome (blue) vs around ISs (red), shown by IS family.** Statistical significance was computed using a Chi2 test. NS: *P* value > 0.05. *: *P* value < 0.05. **: *P* value < 0.01. ***: *P* value < 0.001. ****: *P* value < 0.0001.

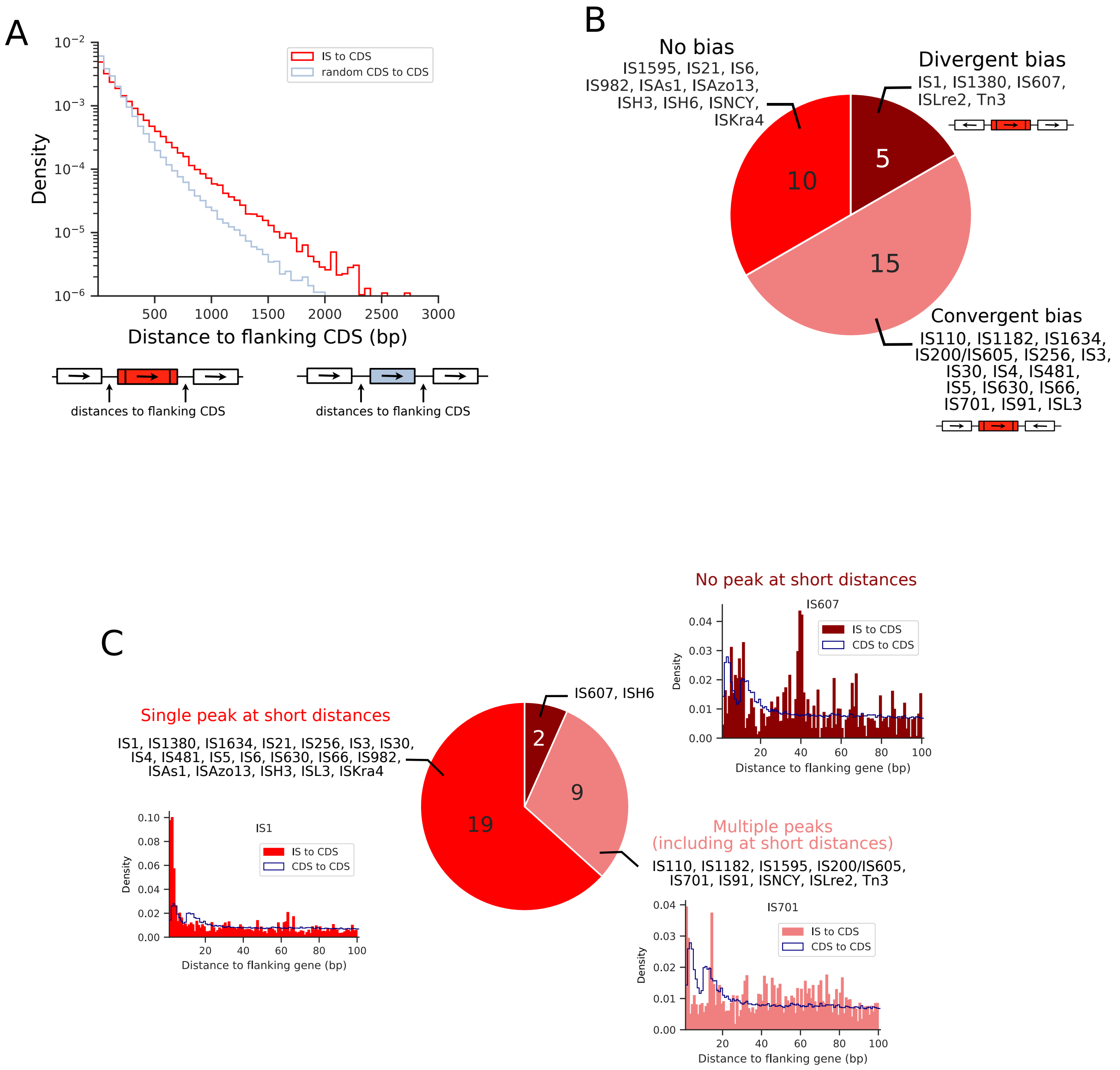

Figure S4. Complementary to Fig. 2. **Distance and orientation properties of IS flanking genes.** A. Normalized distribution of distances to CDSs flanking ISs (red) or random CDSs (light blue). An analysis of IS flanking regions in genomes lacking the IS further suggests that the greater distances observed with ISs stems from original insertions into longer intergenic regions rather than from post-insertion elongation (Fig. S20). B. Distribution of IS families showing no bias in flanking gene orientation (red), a bias toward divergent flanking genes (dark red), or a bias toward convergent flanking genes (pink). C. The curves show distributions of distances between ISs and the flanking CDSs (red) or between random pairs of neighbor CDSs (blue). The pie chart indicates the IS families showing a single peak at short distances (1–5 bp) (red), multiple peaks including at short distances (pink), or no peak at short distances (dark red), with an example distribution for each category.

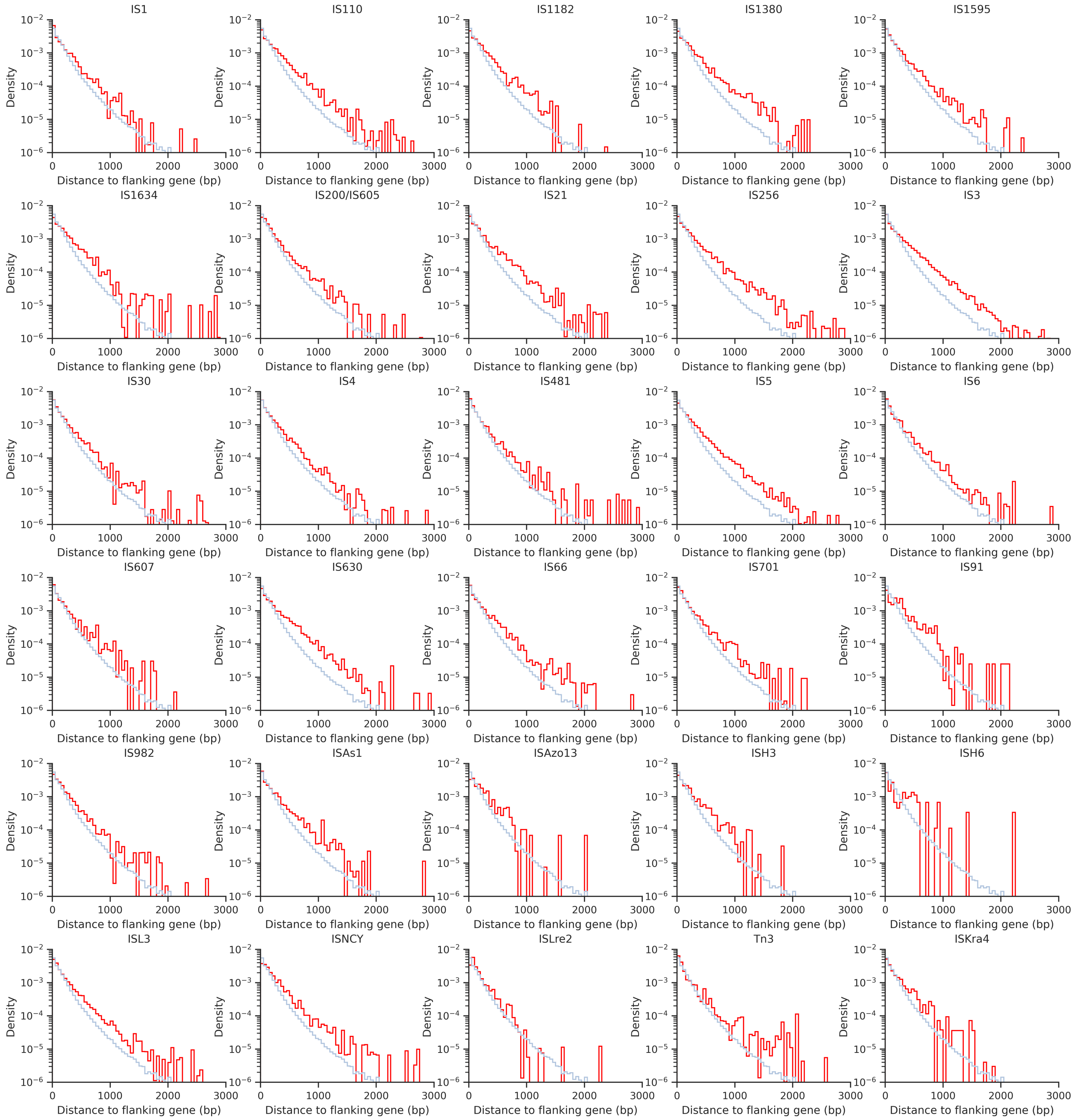

Figure S5. Complementary to Fig. S4A. **Normalized histogram of distances to flanking genes for random CDSs (blue) and ISs (red), shown by IS family.**

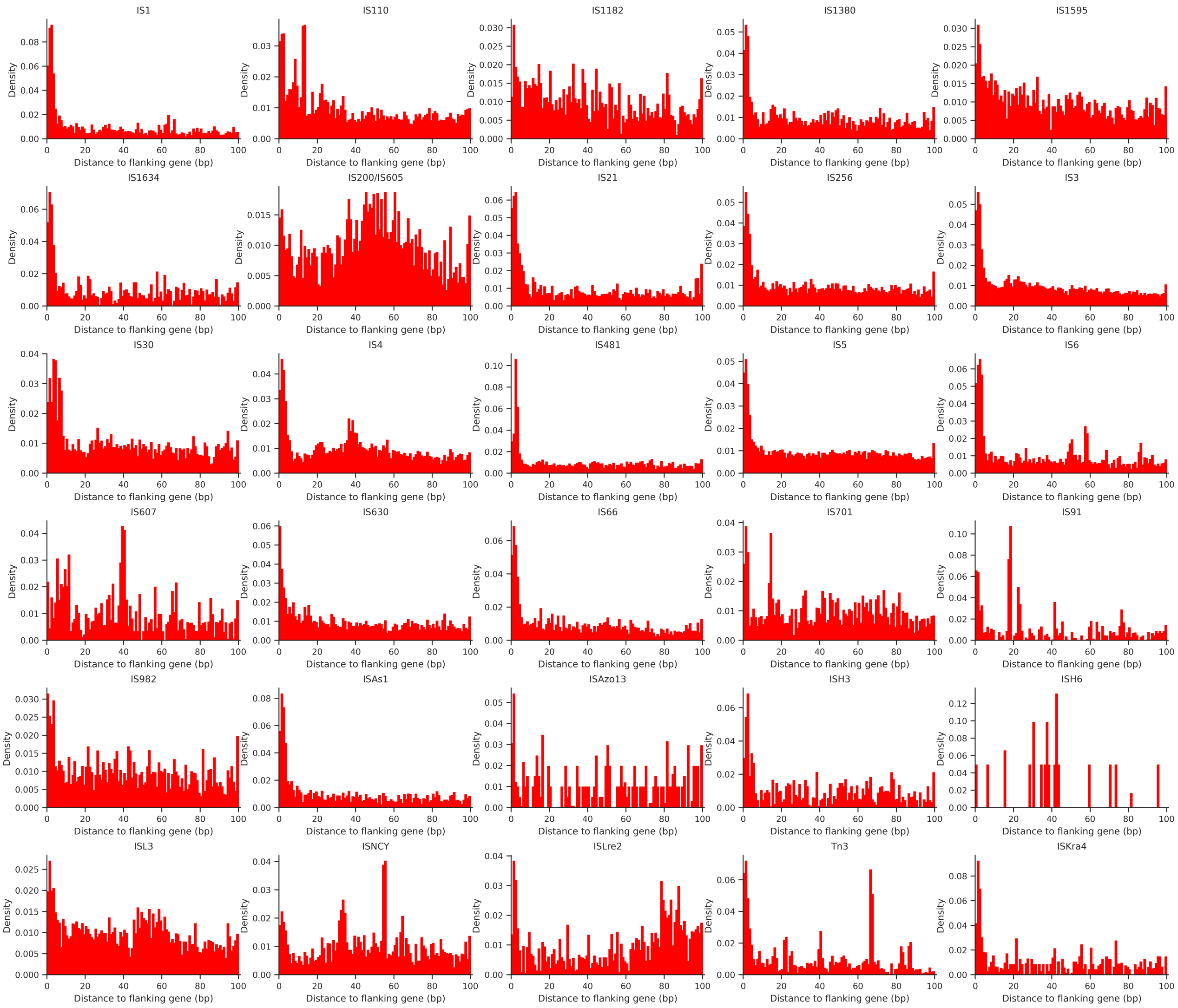

Figure S6. Complementary to Fig. 2E. **Normalized histogram of distances below 100 bp to flanking genes of ISs, shown by IS family.**

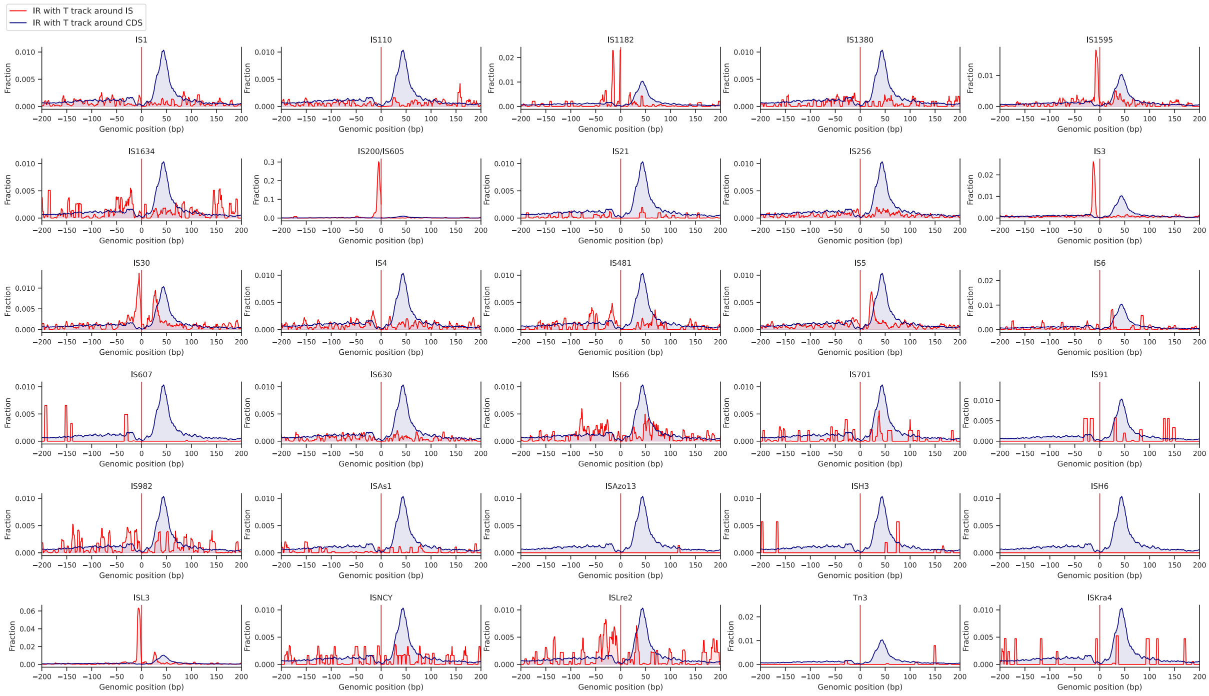

Figure S7. Complementary to Fig. 3. **Frequency of terminator-like sequences in the 200 bp upstream and downstream of IS (red) and gene CDSs (blue) shown by IS family.**

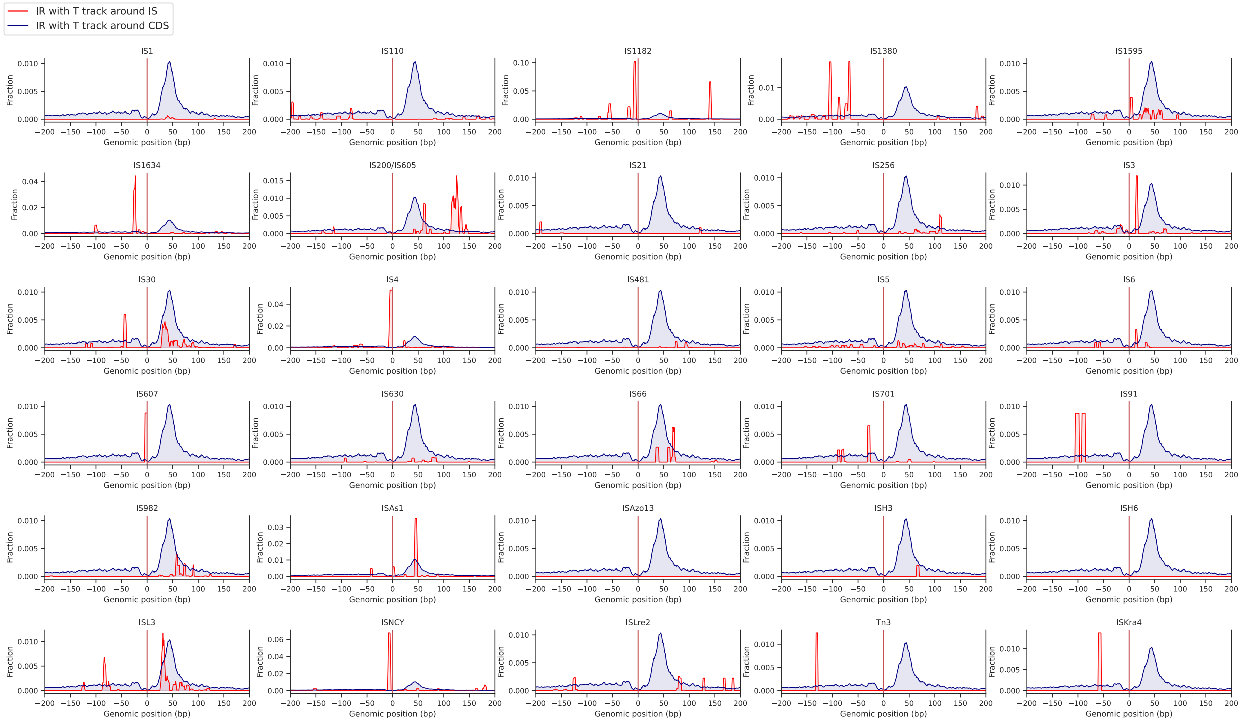

Figure S8. Complementary to Fig. 3. **Frequency of terminator-like sequences found inside of IS sequences (between the limits of the IS and the CDS) (red) and around gene CDSs (blue) shown by IS family.**

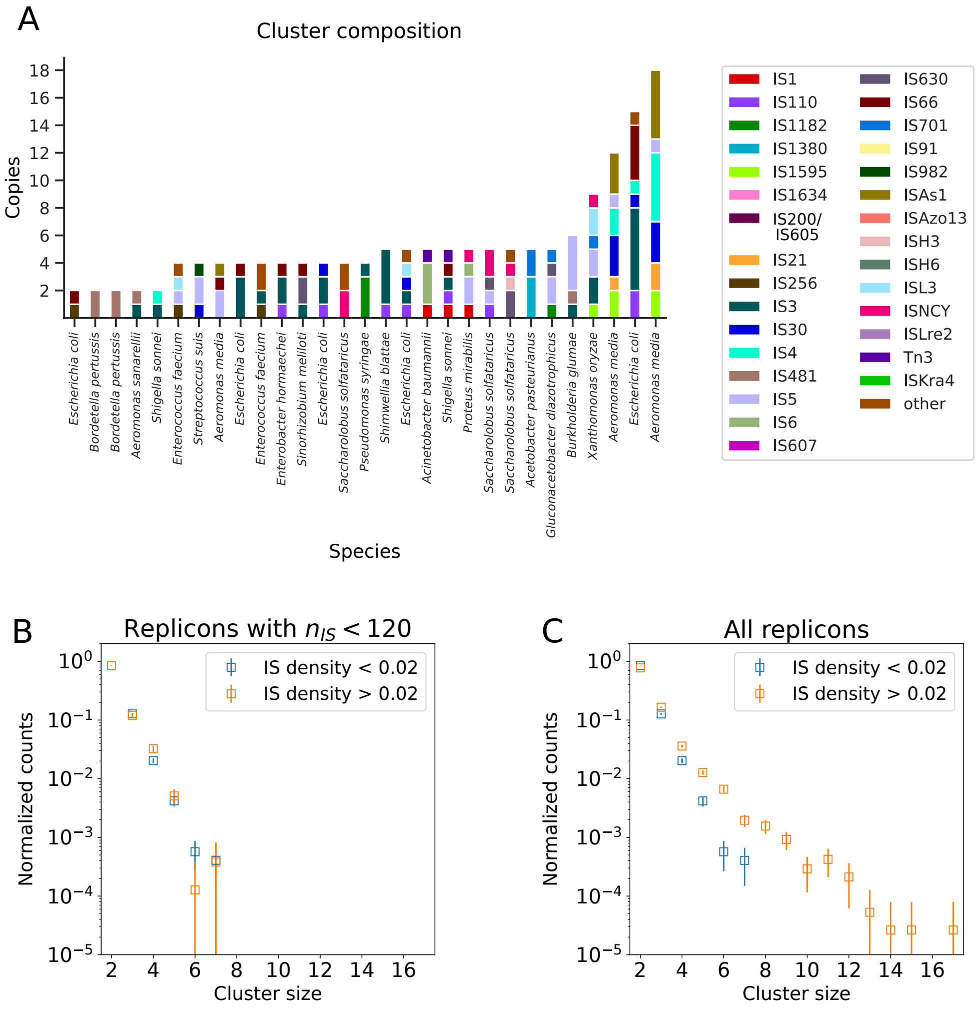

Figure S9. Complementary to Fig. 4. **IS clusters: diversity and statistical properties.** A. Example of cluster compositions for clusters of different sizes coming from different organisms. B,C. IS cluster size distributions for B) replicons containing fewer than 120 ISs and C) all replicons. The data are shown by separating replicons according to their IS density, defined as the number of ISs divided by the number of genes in the replicon.

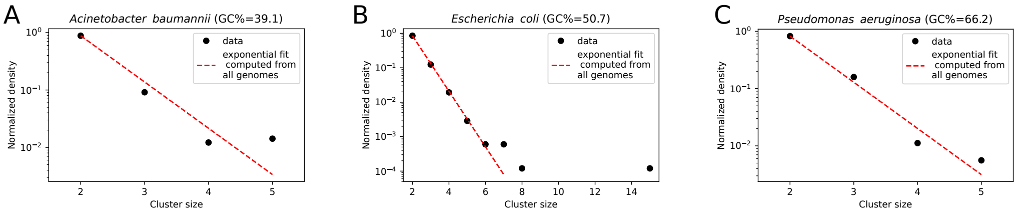

Figure S10. Complementary to Fig. 4. **IS cluster size distributions computed over the genomes of three species.** Left) *A. baumannii*, GC%=39.1. Center) *E. coli*, GC%=50.7. Right) *P. aeruginosa*, GC%=66.2. The red dashed lines represent the exponential fit obtained by considering the entire set of genomes in our datasets (8,106 species). The apparent differences of slope are due to different ranges of the x-axis. The different x-axis ranges mainly reflect the larger number of clusters in *E. coli*(due to more chromosomes), along with one outlier containing 15 ISs.

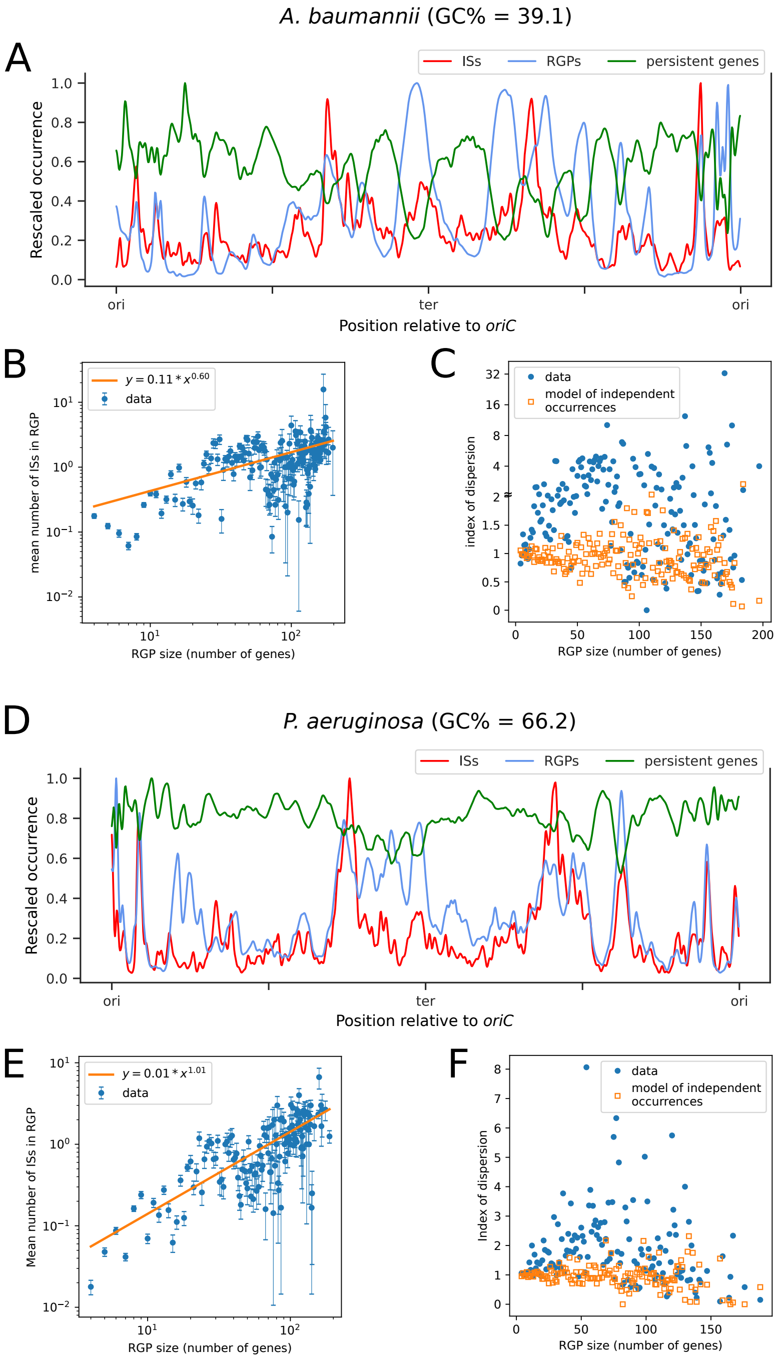

Figure S11. Complementary to Fig. 5. **ISs versus RGPs.** We show for *A. baumannii* (top) and *P. aeruginosa* (bottom) the same plots as in Fig. 5 obtained in *E. coli*. A,C) Distribution of unique IS positions (red), persistent gene positions (green) and frequency at which a region is a region of genomic plasticity (RGP, blue) across chromosomes. B,D) Mean number of IS copies as a function of RGP size (number of genes within each RGP), computed from all RGPs found in the chromosomes of each species, using a log-log scale – error bars represent the standard error of the mean. The orange line indicates the best power-law fit, revealing as in *E. coli* an almost linear relationship in *P. aeruginosa*. C,E) Index of dispersion (i.e., ratio of the variance to the mean) for the IS content of RGPs as a function of RGP size. The orange symbols represent values obtained in a model in which, within a given RGP, genes have a fixed probability of being an IS, independent of other IS occurrences. These probabilities are determined by the fits in (B,D) and are in average equal to $\simeq 1/64$ for *A. baumannii*, and $\simeq 1/70$ for *P. aeruginosa*.

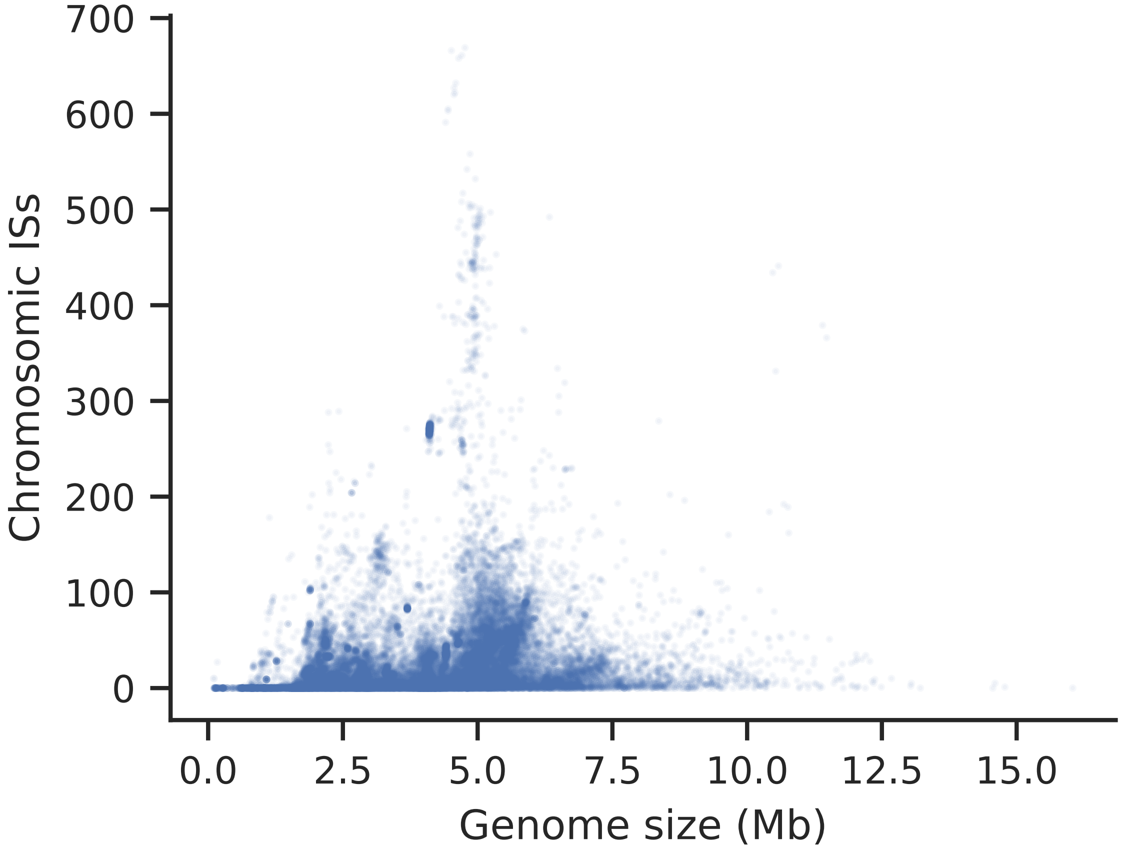

Figure S12. Chromosome size (in Mb) versus total number of ISs found on the chromosome.

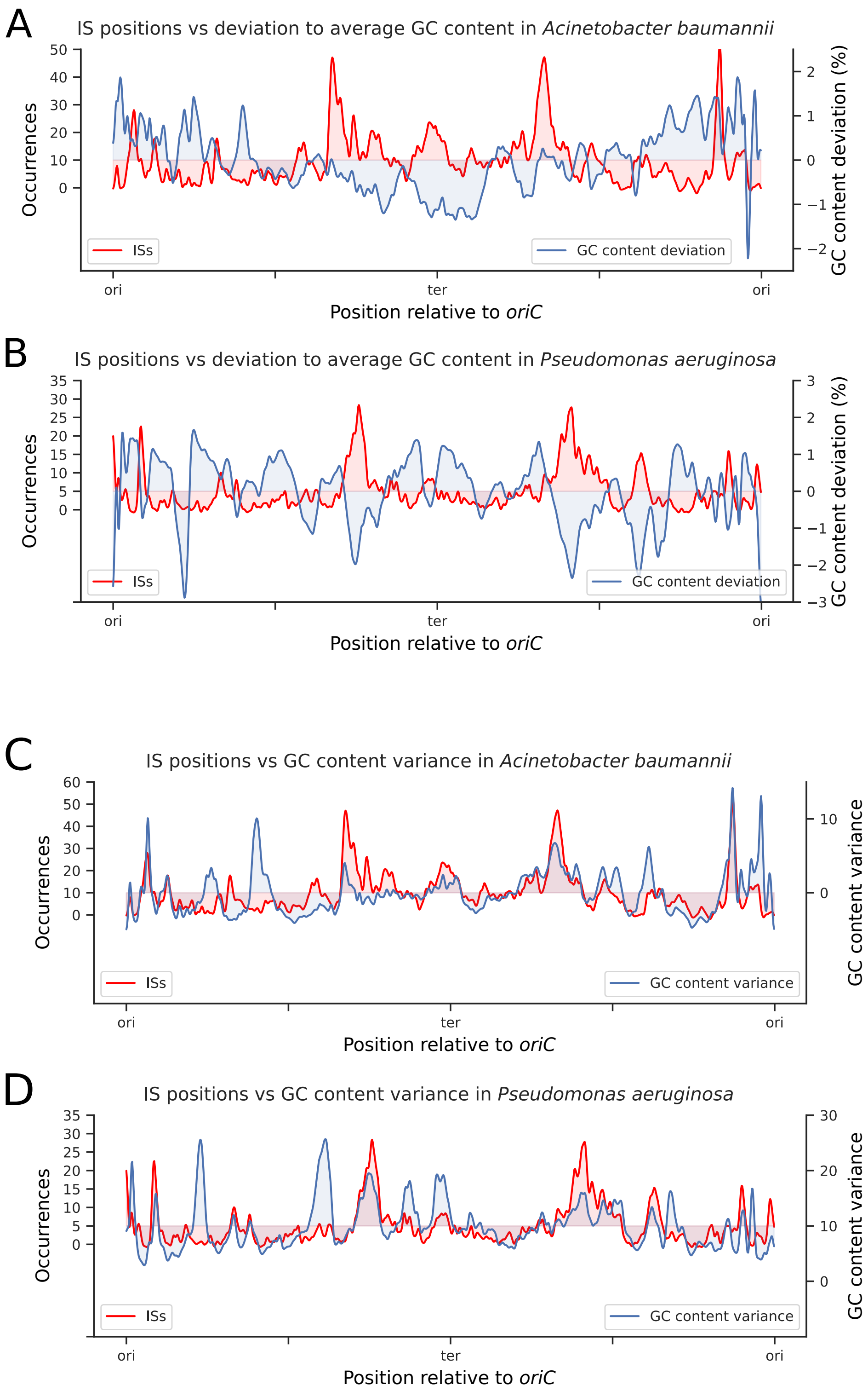

Figure S13. Complementary to Fig. 6. **Characterization of the GC content of IS niches in *A. baumannii* and *P. aeruginosa* genomes.** A,C) Distribution of unique IS positions (red) and deviation to average GC content (blue) across chromosomes. Pearson correlation: -0.20 for *A. baumannii* and -0.51 for *P. aeruginosa*. B, D) Comparison of IS distribution to the variance in GC content across chromosomes. Pearson correlation: 0.45 for *A. baumannii* and 0.40 for *P. aeruginosa*.

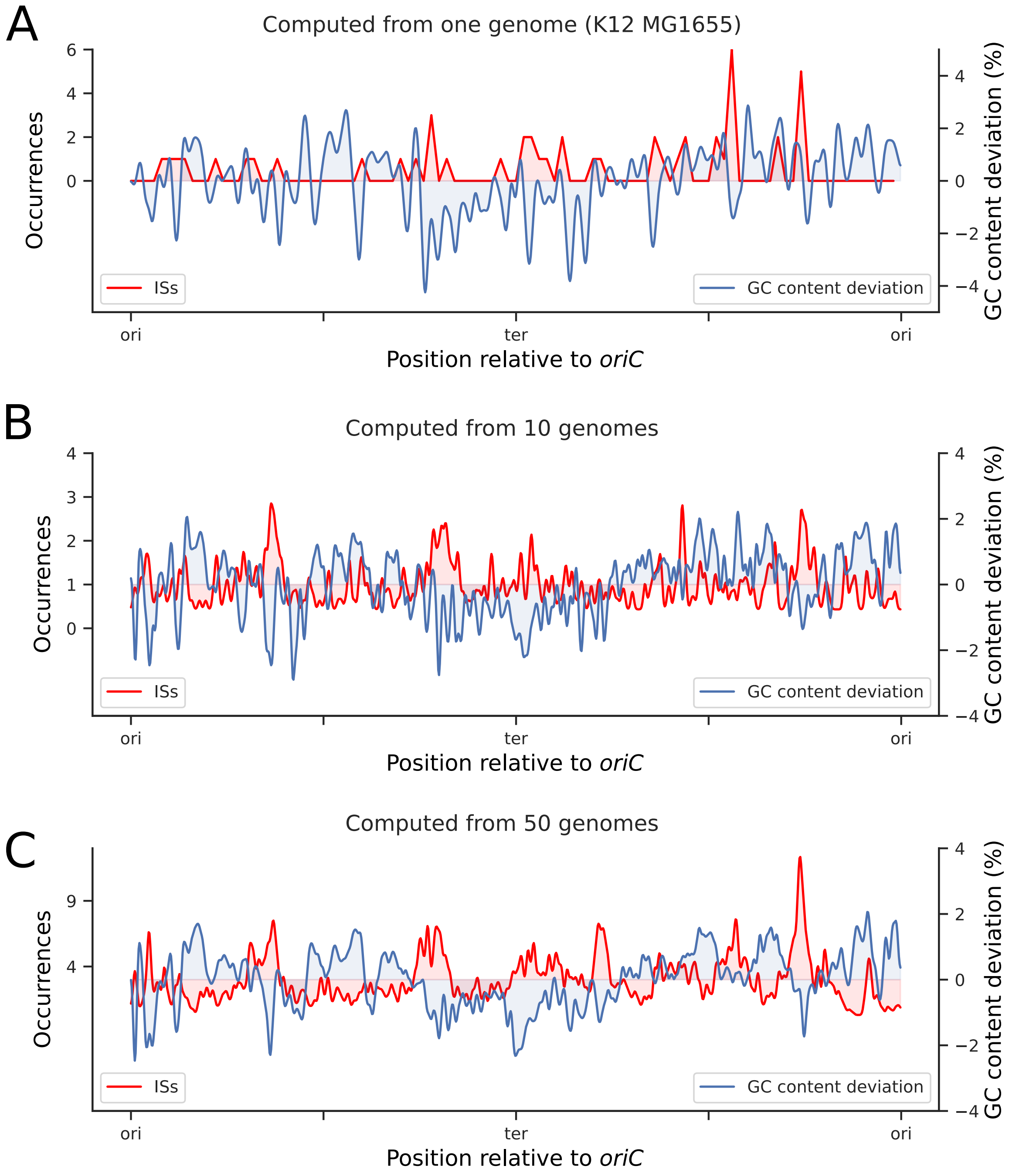

Figure S14. Complementary to Fig. 6. **Genomic distribution of ISs versus deviation from average GC content computed by considering different numbers of genomes.** A) a single genome (*E. coli* K12 MG1655), B) 10 genomes, and C) 50 genomes randomly selected from a set of 1,917 *E. coli* genomes.

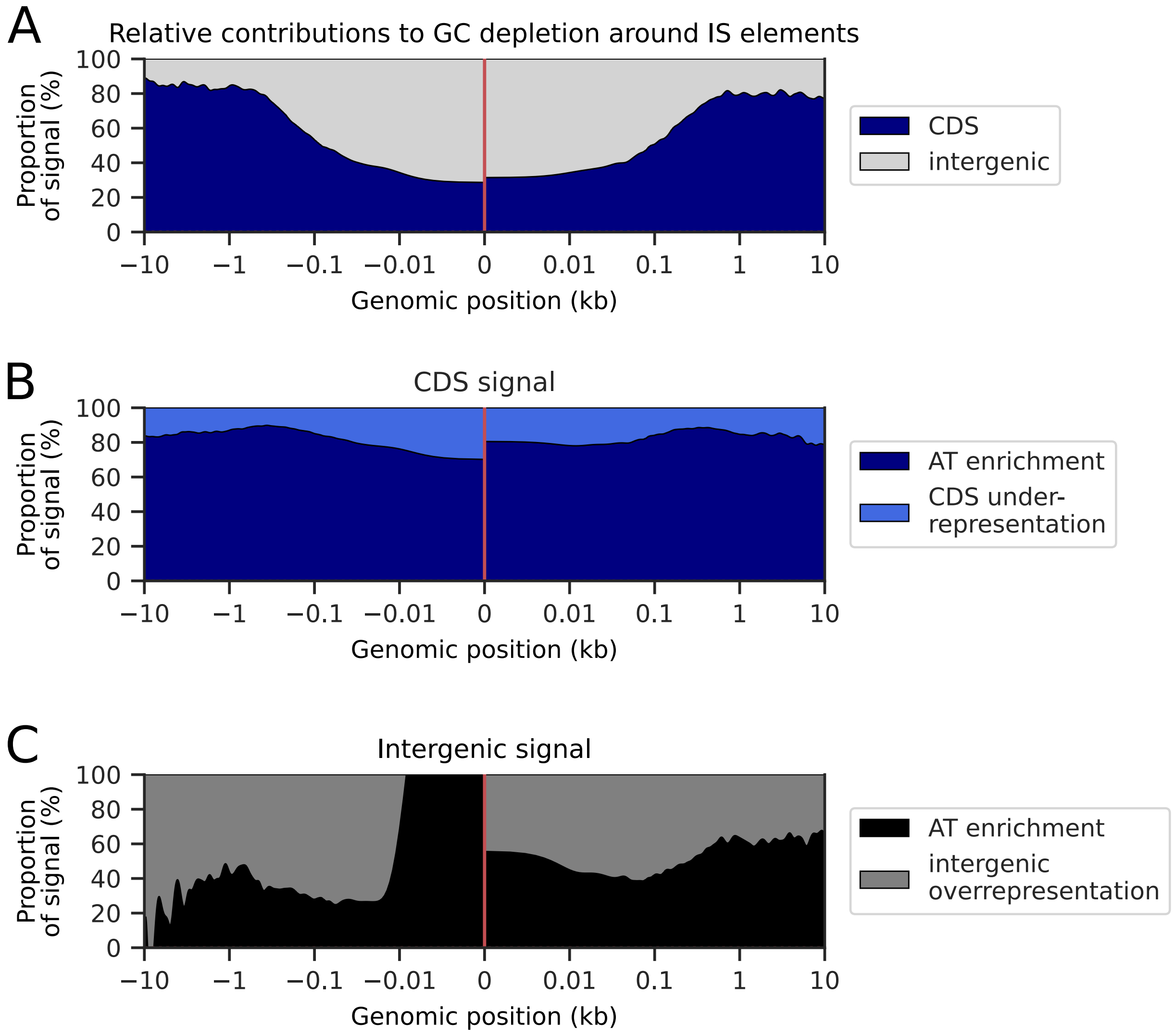

Figure S15. Complementary to Fig. 7. **Analysis of the contributions to the GC depletion around ISs.** A. Relative contributions of CDSs and intergenic sequences to the GC depletion around ISs. B/C. Contribution of AT enrichment and under/overrepresentation to the AT enrichment signal in CDS/intergenic sequences.

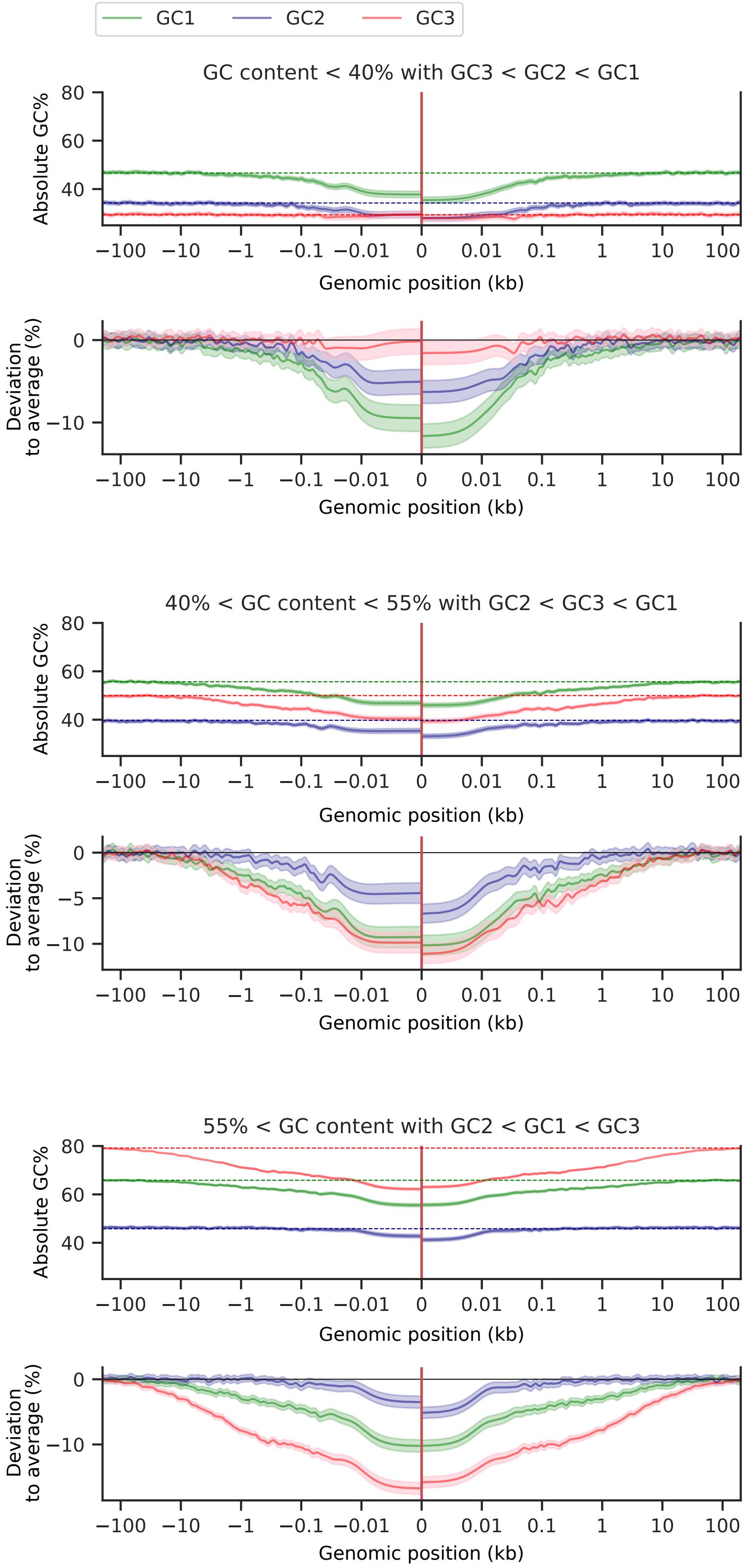

Figure S16. Complementary to Fig. 7. **Deviations from average GC content for each codon position.** We consider three groups of genomes (4; 5): low, medium, and high GC content, from top to bottom. In each case, the first panel shows the average absolute GC content for each codon position, and the second panel shows the corresponding deviation relative to the average GC content for the associated codon position. Number of genomes: 8,984, 10,542, and 10,973 for low, medium, and high GC content, respectively.

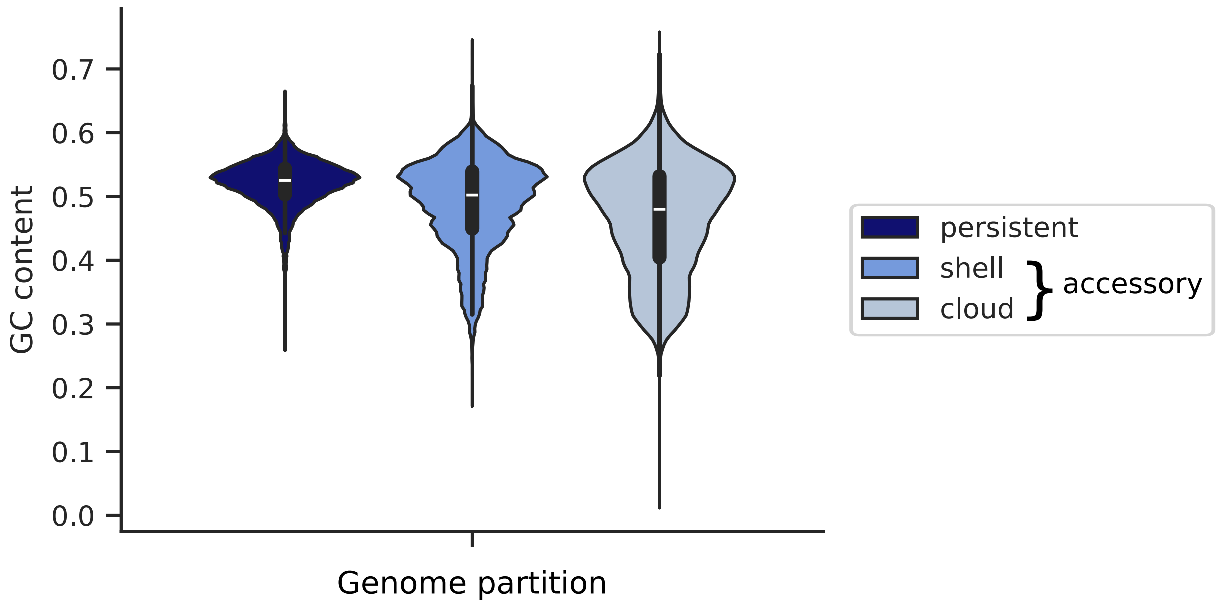

Figure S17. GC content in persistent, shell, and cloud genes in *E. coli* genomes.

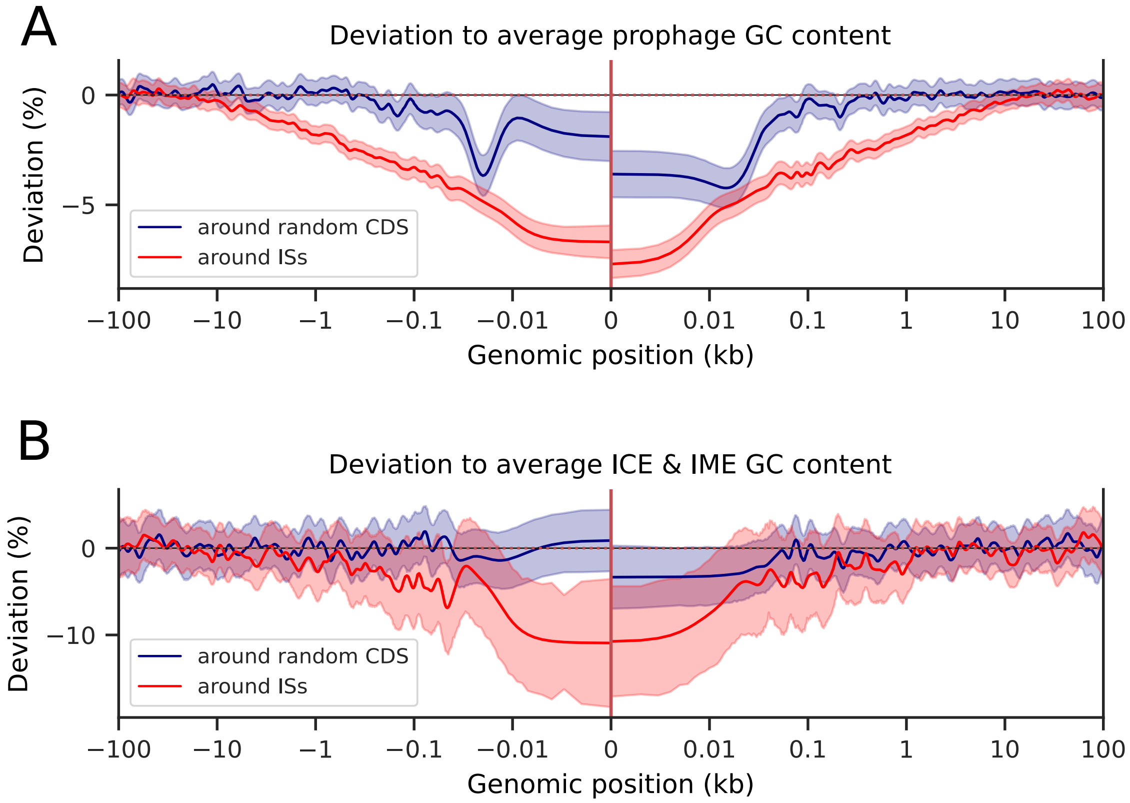

Figure S18. Complementary to Fig. 7. **GC content deviation in MGEs around ISs.** A. GC content deviation in prophage CDSs around ISs and around random CDSs. B. GC content deviation in ICE and IME sequences around ISs and around random CDSs.

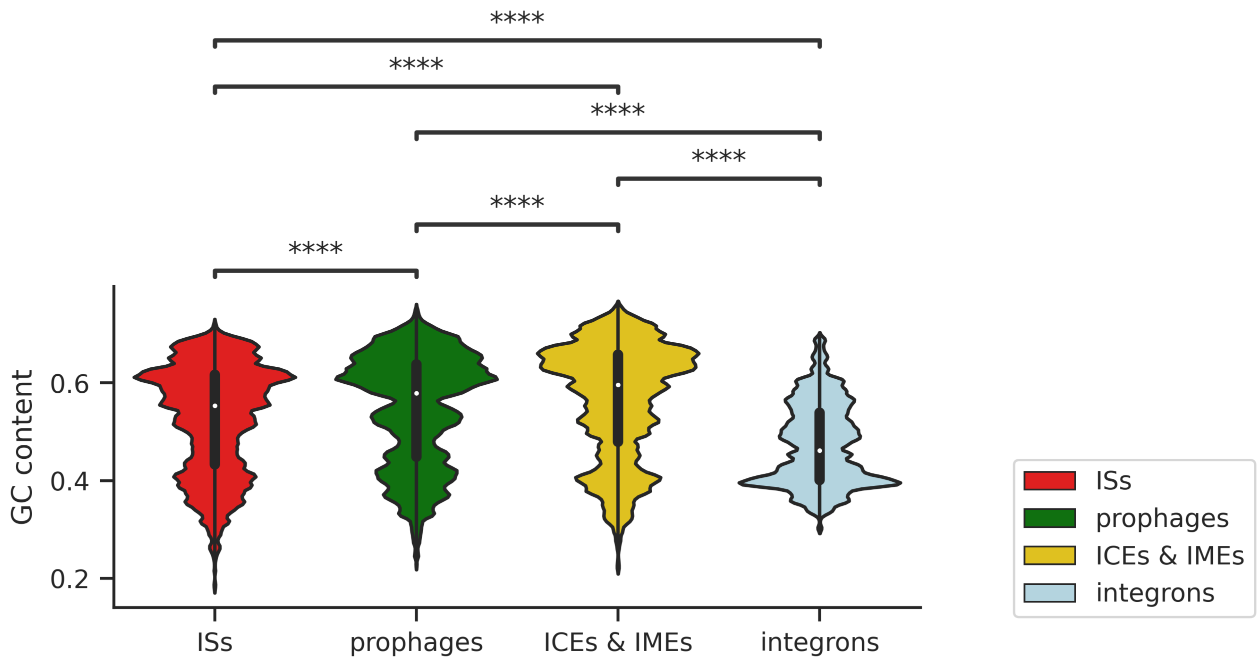

Figure S19. Complementary to Fig. 8. **GC content in ISs, prophages, ICEs/IMEs, and integrons.** Statistical tests: Mann-Whitney-Wilcoxon test two-sided with Bonferroni correction. All *P* values $P < 3e^{-308}$.

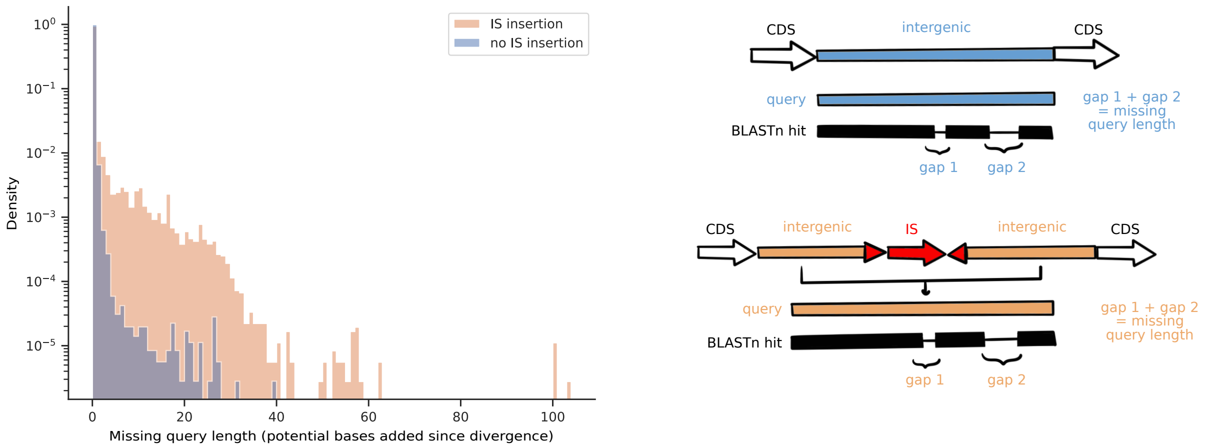

Figure S20. Complementary to Fig. S4A. **Analysis of sequences flanking ISs.** Red: Missing query length when BLASTing sequences around ISs against the same uninterrupted sequences in other genomes (length of bases present around the IS but not in the sequence found with no IS). Blue: missing query length when BLASTing random sequences with no IS within it against a match in other genomes (control).

Supplementary Note 5: Supplementary tables

Supp. Table 1.csv: For each IS family, proportion of copies with a terminator sequence upstream of the ORF inside of the element, upstream of the ORF outside of the element, downstream of the ORF inside of the element, downstream of the ORF outside of the element. For each IS family, a chi2 test was performed to determine if upstream (downstream) terminators were significantly more frequent inside or outside of the element.

Supp. Table 2.csv: List of COG pairs identified as being in a synteny relationship using a false discovery rate of 0.05. See methodology in (6) for details on the method used to identify these pairs.

Bibliography

1. Cerveau, N., Leclercq, S., Leroy, E., Bouchon, D. & Cordaux, R. Short- and long-term evolutionary dynamics of bacterial insertion sequences: Insights from *Wolbachia* endosymbionts. *Genome Biology and Evolution* **3**, 1175–1186 (2011). URL https://academic.oup.com/gbe/article/doi/10.1093/gbe/evr096/595248.
2. Xie, Z. & Tang, H. ISEScan: automated identification of insertion sequence elements in prokaryotic genomes. *Bioinformatics* **33**, 3340–3347 (2017). URL https://academic.oup.com/bioinformatics/article/33/21/3340/3930124.
3. Siguier, P. ISfinder: the reference centre for bacterial insertion sequences. *Nucleic Acids Research* **34**, D32–D36 (2006). URL https://academic.oup.com/nar/article-lookup/doi/10.1093/nar/gkj014.
4. Muto, A. & Osawa, S. The guanine and cytosine content of genomic DNA and bacterial evolution. *Proceedings of the National Academy of Sciences* **84**, 166–169 (1987). URL https://pnas.org/doi/full/10.1073/pnas.84.1.166.
5. Brocchieri, L. The GC content of bacterial genomes. *Journal of Phylogenetics & Evolutionary Biology* **02** (2014). URL http://esciencecentral.org/journals/the-gc-content-of-bacterial-genomes-2329-9002-2-e108.php?aid=26236.
6. Junier, I. & Rivoire, O. Conserved units of co-expression in bacterial genomes: An evolutionary insight into transcriptional regulation. *PLOS ONE* **11**, e0155740 (2016). URL https://dx.plos.org/10.1371/journal.pone.0155740.
